# Candidate *CSPG4* mutations and induced pluripotent stem cell modeling implicate oligodendrocyte progenitor cell dysfunction in familial schizophrenia

**DOI:** 10.1038/s41380-017-0004-2

**Published:** 2018-01-04

**Authors:** Femke M. de Vrij, Christian G. Bouwkamp, Nilhan Gunhanlar, Guy Shpak, Bas Lendemeijer, Maarouf Baghdadi, Shreekara Gopalakrishna, Mehrnaz Ghazvini, Tracy M. Li, Marialuisa Quadri, Simone Olgiati, Guido J. Breedveld, Michiel Coesmans, Edwin Mientjes, Ton de Wit, Frans W. Verheijen, H. Berna Beverloo, Dan Cohen, Rob M. Kok, P. Roberto Bakker, Aviva Nijburg, Annet T. Spijker, P. M. Judith Haffmans, Erik Hoencamp, Veerle Bergink, GROUP Study Consortium, Jacob A. Vorstman, Timothy Wu, Loes M. Olde Loohuis, Najaf Amin, Carolyn D. Langen, Albert Hofman, Witte J. Hoogendijk, Cornelia M. van Duijn, M. Arfan Ikram, Meike W. Vernooij, Henning Tiemeier, André G. Uitterlinden, Ype Elgersma, Ben Distel, Joost Gribnau, Tonya White, Vincenzo Bonifati, Steven A. Kushner

**Affiliations:** 1 Department of Psychiatry, Erasmus Medical Center, Rotterdam, The Netherlands; 2 Department of Clinical Genetics, Erasmus Medical Center, Rotterdam, The Netherlands; 3 Department of Developmental Biology, and Erasmus MC iPS Facility, Erasmus Medical Center, Rotterdam, The Netherlands; 4 Delta Psychiatric Center, Poortugaal, The Netherlands; 5 Department of Neuroscience, Erasmus Medical Center, Rotterdam, The Netherlands; 6 ENCORE Expertise Center for Neurodevelopmental Disorders, Erasmus Medical Center, Rotterdam, The Netherlands; 7 Mental Health Care Organization North-Holland North, Heerhugowaard, The Netherlands; 8 Parnassia Psychiatric Institute, The Hague, The Netherlands; 9 Department of Psychiatry and Psychology, School of Mental Health and Neuroscience, Maastricht University Medical Center, Maastricht, The Netherlands; 10 Psychiatric Center GGZ Centraal, Amersfoort, The Netherlands; 11 Faculty of Social and Behavioral Sciences Clinical, Health and Neuro Psychology, Department of Affective Disorders, PsyQ, Leiden University, Leiden, The Netherlands; 12 Institute of Psychology, Leiden University, Leiden, The Netherlands; 13 Department of Psychiatry, Brain Center Rudolf Magnus, University Medical Center Utrecht, Utrecht, The Netherlands; 14 Department of Psychiatry, The Hospital for Sick Children and University of Toronto, Toronto, Ontario Canada; 15 Program in Genetics and Genome Biology, Research Institute, The Hospital for Sick Children, Toronto, Ontario Canada; 16 Center for Neurobehavioral Genetics, Semel Institute for Neuroscience and Human Behavior, University of California, Los Angeles, CA USA; 17 Department of Epidemiology, Erasmus Medical Center, Rotterdam, The Netherlands; 18 Department of Radiology, Erasmus Medical Center, Rotterdam, The Netherlands; 19 Department of Medical Informatics, Erasmus Medical Center, Rotterdam, The Netherlands; 20 Biomedical Imaging Group Rotterdam, Departments of Radiology & Medical Informatics, Erasmus Medical Center, Rotterdam, The Netherlands; 21 Department of Neurology, Erasmus Medical Center, Rotterdam, The Netherlands; 22 Department of Internal Medicine, Erasmus Medical Center, Rotterdam, The Netherlands; 23 Department of Medical Biochemistry, Academic Medical Centre, Amsterdam, The Netherlands

**Keywords:** Genetics, Stem cells, Schizophrenia

## Abstract

Schizophrenia is highly heritable, yet its underlying pathophysiology remains largely unknown. Among the most well-replicated findings in neurobiological studies of schizophrenia are deficits in myelination and white matter integrity; however, direct etiological genetic and cellular evidence has thus far been lacking. Here, we implement a family-based approach for genetic discovery in schizophrenia combined with functional analysis using induced pluripotent stem cells (iPSCs). We observed familial segregation of two rare missense mutations in *Chondroitin Sulfate Proteoglycan 4* (*CSPG4*) (c.391G > A [p.A131T], MAF 7.79 × 10^−5^ and c.2702T > G [p.V901G], MAF 2.51 × 10^−3^). The *CSPG4*^*A131T*^ mutation was absent from the Swedish Schizophrenia Exome Sequencing Study (2536 cases, 2543 controls), while the *CSPG4*^*V901G*^ mutation was nominally enriched in cases (11 cases vs. 3 controls, *P* = 0.026, OR 3.77, 95% CI 1.05–13.52). CSPG4/NG2 is a hallmark protein of oligodendrocyte progenitor cells (OPCs). iPSC-derived OPCs from *CSPG4*^*A131T*^ mutation carriers exhibited abnormal post-translational processing (*P* = 0.029), subcellular localization of mutant NG2 (*P* = 0.007), as well as aberrant cellular morphology (*P* = 3.0 × 10^−8^), viability (*P* = 8.9 × 10^−7^), and myelination potential (*P* = 0.038). Moreover, transfection of healthy non-carrier sibling OPCs confirmed a pathogenic effect on cell survival of both the *CSPG4*^*A131T*^ (*P* = 0.006) and *CSPG4*^*V901G*^ (*P* = 3.4 × 10^−4^) mutations. Finally, *in vivo* diffusion tensor imaging of *CSPG4*^*A131T*^ mutation carriers demonstrated a reduction of brain white matter integrity compared to unaffected sibling and matched general population controls (*P* = 2.2 × 10^−5^). Together, our findings provide a convergence of genetic and functional evidence to implicate OPC dysfunction as a candidate pathophysiological mechanism of familial schizophrenia.

## Introduction

Schizophrenia is a severely debilitating psychiatric disorder affecting ~1% of the population worldwide [1]. The strongest known determinant for developing schizophrenia is family history. A meta-analysis, which included five decades of twin studies, concluded a heritability estimate (*h*^2^) of 0.77 ± 0.05, with a relatively limited contribution of shared environmental influences (*c*^2^) (0.013 ± 0.025) [2].

The Psychiatric Genomics Consortium recently reported a genome-wide association study (GWAS) investigating 36,989 cases and 113,075 controls, in which 128 genome-wide significant single-nucleotide variants were identified across 108 independent genomic loci, suggesting an important contribution of common genetic variation to schizophrenia risk [3]. However, a large proportion of the heritability for schizophrenia remains unexplained, leaving many genetic variants remaining to be discovered. Therefore, increasing attention has also been focused on the potential contribution of rare genomic variation to schizophrenia risk. Copy number variants (CNVs) are a well-established source of pleiotropic risk, ranging from asymptomatic carriership to a complex constellation of symptoms affecting multiple organ systems, such as the 22q11.2 microdeletion syndrome [4]. In a large-scale schizophrenia case-control cohort analysis, known pathogenic CNVs were significantly more frequent in cases (2%) than controls (0.4%) [5, 6]. Moreover, an independent study found that large (>500 kb) CNVs are enriched in loci associated with schizophrenia by GWAS and frequently involve genes encoding proteins located in the postsynaptic density [7].

In addition to microarray-based genotyping methods, the development of next-generation sequencing has allowed the possibility to examine whether rare single nucleotide variants or small insertions-deletions contribute to schizophrenia risk. A Swedish cohort including 2536 cases and 2543 controls yielded no single mutation or single gene reaching genome-wide significance for association with schizophrenia, but confirmed a similar enrichment of gene sets for synaptic function as previously identified for genes located in schizophrenia-associated CNVs and GWAS loci [8]. Moreover, trio-based studies have identified a number of candidate genes through identification of recurrent de novo mutations [9, 10] and an increased burden of mutations occurring in genes encoding glutamatergic postsynaptic proteins [11].

Recent genetic and induced pluripotent stem cell (iPSC)-based studies have converged on a model by which neuronal function, and in particular synaptic transmission, is a major pathophysiological mechanism of schizophrenia [3, 8, 11–13]. However, functional neuronal alterations may arise either by direct cell-type autonomous changes to neurons themselves, or indirectly through a primary pathophysiological influence on other cell types that influence neuronal function. Numerous studies have reported the involvement of glial cell biology in the pathophysiology of schizophrenia, including alterations in oligodendrocytes, myelination, and white matter integrity [14–19], which directly regulate neuronal function.

Abnormalities of the integrity of the white matter are strongly associated with schizophrenia [20]. The late adolescent critical period for cerebral cortex myelination has long been recognized as overlapping closely with the typical age of onset for schizophrenia [16, 17]. Myelination-related genes have been shown to be enriched for common variants associated independently to white matter integrity [21] and schizophrenia [15, 22]. Two recent brain imaging studies have elegantly compared white matter integrity in 16p11.2 deletion and duplication carriers [23, 24] of which only the 16p11.2 duplication confers increased risk for schizophrenia [6]. Notably, both global fractional anisotropy (FA) and white matter volume were selectively decreased in 16p11.2 duplication carriers. However, despite increasing evidence of an association between schizophrenia and myelination integrity, the molecular and cellular mechanisms by which oligodendrocyte lineage dysfunction might influence schizophrenia risk have remained largely unknown.

We now report genetic and functional evidence of oligodendrocyte progenitor cell dysfunction in schizophrenia. Using a family-based genetic approach, we observed multiple rare missense mutations in *CSPG4* that segregate with schizophrenia. Functional studies using iPSCs reprogrammed from affected *CSPG4* mutation carriers and their unaffected non-carrier siblings revealed that patient-derived OPCs exhibit abnormal post-translational processing, aberrant subcellular localization of CSPG4/NG2, abnormal cellular morphology, reduced cellular viability, and impaired oligodendrogenesis. Moreover, diffusion tensor imaging (DTI) of *CSPG4* mutation carriers confirmed a global impairment in white matter integrity, together providing support for OPC dysfunction as a candidate pathophysiological mechanism of schizophrenia.

## Methods summary

### Genetic analysis

Linkage and copy number analysis was performed with Illumina HumanCytoSNP-12v2 chip arrays using an affected-only model with an assumption of 99.9% penetrance. Analysis revealed a total of 294.34 Mb of genomic regions, with suggestive linkage on chromosomes 2, 11, 14, 15, and 16. Whole-genome exome sequencing was performed twice: initially at 40×, and again at 90× coverage. Exome variants were considered for additional validation if they were rare (minor allele frequency (MAF) < 0.001), predicted to alter coding sequence (missense, nonsense, frameshift, essential splice site), and occurred within the regions of suggestive linkage.

### Cellular studies

Human iPSCs were differentiated to neural progenitor cells (NPCs) and neurons by embryoid body-based neural differentiation [25]. Electrophysiology was performed in whole-cell patch-clamp configuration after 8–10 weeks of differentiation. iPSC-derived OPCs were differentiated according to Monaco et al. [26] with modifications. Biotinylation of cell surface proteins was adapted from Huang et al. [27]. OPC viability was assessed by quantitative fluorometric monitoring of resazurin conversion to resorufin [28]. Myelination assay was performed using *ex vivo* organotypic cerebral cortex slices of Shiverer mice, as previously described [29, 30].

### Magnetic resonance imaging (MRI)

Two patients and one control sibling of the discovery family were subjected to MRI scanning. Population controls (*n* = 294) matched on age, gender, and pack-years of cigarette smoking were selected from the Rotterdam Study. MRI images were obtained using a 1.5 Tesla General Electric (GE Healthcare, Milwaukee, Wisconsin, USA) MR system using a bilateral phased-array head coil. A full description of the imaging protocol and Rotterdam Study design has been described elsewhere [31]. An in-house MATLAB (Mathworks, Natick, MA) program was used to quantify the number and spatial characteristics of white matter “potholes” along the major white matter tracts [32].

## Results

### Genetic findings in the discovery family

**Fig. 1:**
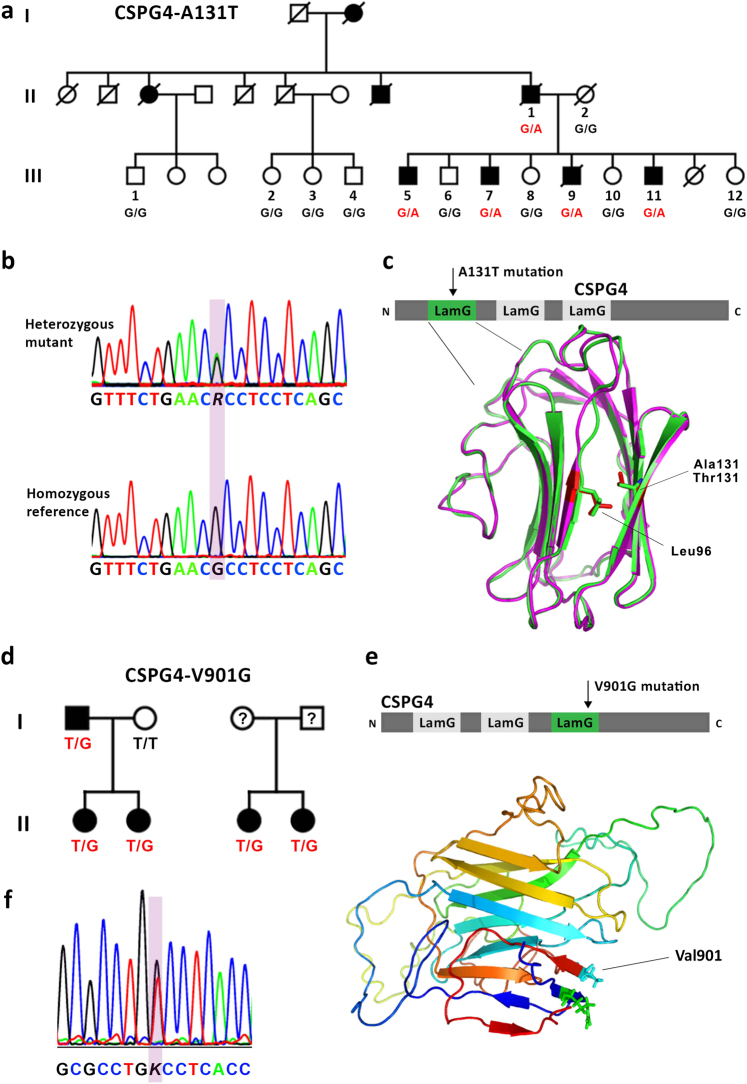
Familial segregation of *CSPG4* mutations with schizophrenia. **a** Pedigree of multiplex discovery family with schizophrenia. Symbols: filled, schizophrenia; open, unaffected; G/A, heterozygous carrier of the *CSPG4* c.391G > A mutation (*CSPG4*^*A131T*^); G/G, homozygous reference. **b** Representative sequencing results for heterozygous carriers of the *CSPG4* c.391G > A mutation. The lower panel reflects homozygous reference sequence. **c** Homology model of the first Laminin G domain of CSPG4. Structural alignment of the reference (green) and mutant model (magenta) reveals a difference in the predicted interaction between amino acid positions 131 and 96 (Leu) in the opposing β-strand inside the hydrophobic core of the β-sandwich (predicted alterations of the side chains in red). **d** Family pedigrees segregating the *CSPG4* c.2702T > G mutation (*CSPG4*^*V901G*^). Symbols: filled, schizophrenia; open, unaffected; T/G, heterozygous carrier of the *CSPG4* c.2702T > G mutation; T/T, homozygous reference. **e** Three-dimensional structural homology modeling of the putative 3rd LamG domain of CSPG4 (a.a. 634–921), demonstrating the outside surface location of Val^901^. **f** Representative Sanger sequencing trace of heterozygous carrier of the *CSPG4* c.2702T > G mutation

A non-consanguineous family of Dutch ancestry was ascertained with a pattern of schizophrenia inheritance compatible with autosomal dominant transmission. The core pedigree consisted of a couple and their nine children (five males, four females) of whom the father and four sons suffered from non-syndromic schizophrenia (Fig. 1a and Supplementary Table 1).

Genome-wide parametric linkage analysis was performed on peripheral blood DNA using an autosomal-dominant, affected-only model of inheritance, in order to identify genomic regions shared among all affected family members (Supplementary Table 2). Whole exome sequencing was performed on three individuals of the family (pedigree IDs: II-2, III-5, and III-9; Fig. 1a). Five candidate heterozygous variants were identified based on the following criteria: (a) located within the genomic regions shared among all affected family members, (b) predicted to affect protein coding (missense, nonsense, frameshift, splice site), (c) called in at least one of the affected individuals (III-5 and III-9), (d) absent from the unaffected mother (II-2), (e) absent from dbSNP129, and (f) with a MAF < 0.001 in the Exome Aggregation Consortium (ExAC) browser (Europeans non-Finnish) [33], EVS6500 European Americans, NHLBI Exome Sequencing Project (ESP) [34], 1000 Genomes [35], and Genome of the Netherlands (GoNL) [36] cohorts (Supplementary Table 3). Genotyping of these variants was performed by Sanger sequencing in all participating family members.

Among the five candidate variants, *CSPG4* c.391G > A (p.A131T) was the only variant shared by all affected family members and absent in all unaffected relatives, including in the extended family (Fig. 1a). *CSPG4* c.391G > A (p.A131T) is present in the Exome Aggregation Consortium Browser (Total [forward strand]: T = 6/C = 118,148 alleles [MAF 5.08 × 10^−5^], European (Non-Finnish): T = 5/C = 64,215 alleles [MAF 7.79 × 10^−5^]) [33] but absent from the Swedish Schizophrenia Exome Sequencing Study (2536 cases, 2543 controls) [8], 1000 Genomes [35], and GoNL [36].

### Additional genotyping of *CSPG4* discovery family variant

In an effort to further characterize the frequency of *CSPG4* c.391G > A (p.A131T) in the Netherlands, we performed TaqMan genotyping and Sanger sequencing validation in an independent Dutch cohort of 1219 schizophrenia cases and in the general population-based Rotterdam Study cohort [37] (10,611 subjects). One carrier was identified among the schizophrenia cases (MAF_cases_ 4.1 × 10^−4^) and three within the general population (MAF_population_ 1.4 × 10^−4^). The patient carrier had a long history of severe psychiatric illness, including multiple hospital admissions and chronic antipsychotic and antidepressant medication. Her most recent prescriptions included penfluridol oral depot (20 mg/week) and venlafaxine (37.5 mg/day). Family members could not be ascertained for additional psychiatric history or genotyping. Among the three unrelated carriers (maximum pairwise $\hat \pi = 0.078$) identified in the Rotterdam Study general population cohort, two had a clinically significant history of psychiatric illness ($\hat \pi = 0.055$). One of the subjects had a history of multiple inpatient psychiatric hospitalizations for depression, and the other required chronic antidepressant and anxiolytic pharmacotherapy. Notably these findings were unlikely due to chance alone, given the 12.76% period prevalence of antidepressant use in the Rotterdam Study cohort and 0.065% annual prevalence of inpatient psychiatric hospitalization in the Netherlands [38, 39] (Binomial *P* = 8.8 × 10^−3^).

### Identification of a second rare *CSPG4* variant that segregates with schizophrenia

A previous study identified suggestive linkage at chromosome 15q22-24, containing *CSPG4*, in a cohort of 175 families with schizophrenia or schizoaffective disorder of Central American/Hispanic origin [40, 41]. We therefore sequenced the full open reading frame of *CSPG4* in one proband from each of the 73 families that positively contributed to the linkage signal at this locus (markers D15S131 and D15S655) (Supplementary Table 4). Four rare missense variants were identified with MAF < 0.005 (ExAC Browser Latino) (Fig. 1, Supplementary Fig. 1). However in contrast to the other three variants, *CSPG4* c.2702T > G (p.V901G) was found in two independent families, without evidence of incomplete penetrance (Fig. 1d, f), and located within a LamG domain similar to the c.391G > A (p.A131T) discovery family variant (Fig. 1e). Moreover, the c.2702T > G p.V901G variant was nominally enriched in cases from the Swedish Schizophrenia Exome Sequencing Study [8] (MAF_cases_ 2.19 × 10^−3^; MAF_controls_ 5.82 × 10^−4^, Fisher’s Exact Test *P* = 0.026, OR 3.77, 95% CI 1.05–13.52). Together, these findings suggest that rare coding variants of *CSPG4* might influence the risk of familial schizophrenia.

### Structural protein modeling of *CSPG4* mutations

The *CSPG4*^*A131T*^ and *CSPG4*^*V901G*^ mutations are located within the first and third Laminin G domain of the protein encoded by *CSPG4*, known as neural/glial 2 (NG2) (Fig. 1c, e). Laminin G (LamG) domains are highly conserved across a diverse group of extracellular matrix proteins [42]. Intriguingly, several schizophrenia-associated genes such as *NRXN1* and *LAMA2* also contain LamG domains [10, 43–45]. Crystal structures of LamG domains in the Protein Data Bank allowed homology modeling of the LamG domains of NG2. Models implemented using Phyre2 [46] and I-TASSER [47] both suggested that in the reference sequence, Ala^131^ and Leu^96^ interact across opposing β-sheets inside the hydrophobic core of the β-sandwich. The mutation of Ala^131^, which has a small hydrophobic side chain, to Thr^131^, containing a larger polar side chain, suggests a conformational change impairing the proper folding of the β-sandwich (Fig. 1c).

Interestingly, *CSPG4*^*V901G*^ is located in a putative 3^rd^ LamG domain predicted by I-TASSER [47] (a.a. 634–921, Fig. 1e). This region has not previously been annotated as a LamG domain, despite the striking structural homology to other LamG domains with available crystal structures, most notably that of NRXN1 [48–50]. In contrast to the *CSPG4*^*A131T*^ mutation that is located on the inside of the globular structure of the first LamG domain of NG2, the *CSPG4*^*V901G*^ mutation is predicted to be located on the outside of the putative third LamG domain (Fig. 1e), therefore perhaps affecting protein–protein interactions. Intriguingly, the same protein region has been found to bind to collagen V and VI, implicated in cell adhesion and migration of NG2-expressing cells [51, 52].

### Family-based iPSC modeling of the *CSPG4*^*A131T*^ mutation

#### No evidence for a cell-autonomous neuronal phenotype

Recent genetic and iPSC-based studies of schizophrenia have converged on a model by which neuronal function, and in particular synaptic transmission, is a major pathophysiological mechanism [3, 8, 11–13]. We obtained skin biopsies for iPSC reprogramming from three affected *CSPG4*^*A131T*^ carriers and three unaffected non-carriers within the core sibship of the discovery family (Supplementary Fig. 2a–d). Directed differentiation of iPSCs yielded forebrain-specified NPCs uniformly positive for Nestin, SOX2, Vimentin, and FOXG1 (Supplementary Fig. 2e).

**Fig. 2:**
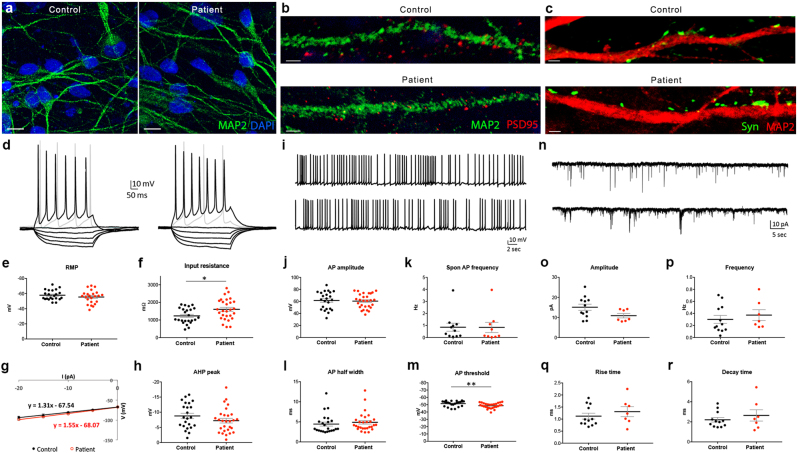
Normal passive, active, and synaptic function in *CSPG4*^*A131T*^ patient iPSC-derived neurons. **a** Immunostaining of iPSC-derived neural cultures after 8 weeks of differentiation (scale bar = 10 µm). **b**, **c** Immunostaining with MAP2, PSD95, and synapsin antibodies confirmed the presence of synaptic proteins on dendrites of iPSC-derived neurons 8 weeks post differentiation (scale bar = 2 µm). **d**–**r** Electrophysiological measurements of iPSC-derived neurons. **d** Representative voltage responses to hyperpolarizing (range: −20−0 pA, 5 pA increments) and depolarizing (10 and 20 pA) current steps (left: control, right: patient). **e** Resting membrane potential (RMP) (*t*_44_ = 1.04, *P* = 0.30). **f** Input resistance (*t*_50_ = 2.54, *P* = 0.01). **g** Current–voltage (I–V) relationship of patient and control cells. **h** AHP peak (*t*_49_ = 1.35, *P* = 0.18). **i** Representative traces of spontaneous action potential (AP) firing (50 s at RMP; top: control, bottom: patient). **j** AP amplitude (*t*_49_ = 0.31, *P* = 0.76). **k** Spontaneous firing rate (*t*_19_ = 0.03, *P* = 0.98). **l** AP half-width (*t*_49_ = 0.65, *P* = 0.52). **m** AP voltage threshold (*t*_49_ = 2.84, *P* = 0.007). **n** Representative traces of spontaneous postsynaptic currents (100 s at −90 mV; top: control, bottom: patient). **o** sPSC amplitude (*t*_17_ = 1.94, *P* = 0.07). **p** sPSC frequency (*t*_17_ = 0.65, *P* = 0.52). **q** sPSC rise time (*t*_17_ = 0.84, *P* = 0.41). **r** sPSC decay time (*t*_17_ = 0.84, *P* = 0.41). **d**–**r** Unpaired two-tailed *t*-test. Passive properties and evoked APs (*N* = 24 control, *N* = 28 patient). Spontaneous APs (*N* = 11 control, *N* = 9 patient). Spontaneous postsynaptic currents (*N* = 12 control, *N* = 7 patient). All error bars are +/− standard errors of the mean (SEM)

NPCs were differentiated to neural cultures for 8–10 weeks, which notably lack cells of the oligodendrocyte lineage including OPCs. Both control and patient-derived neurons developed robust synaptic network connectivity, confirmed by confocal immunofluorescence (Fig. 2a–c) and whole-cell patch-clamp electrophysiological recordings (Fig. 2d–r). Overall, neurons derived from patient carriers and their unaffected siblings had largely similar electrophysiological properties, including passive membrane properties, action potential characteristics, and synaptic physiology. However, two electrophysiological parameters were significantly different between patient and control iPSC-derived neurons—input resistance (control: 1233 ± 88.9 mΩ, patient: 1605 ± 112 mΩ; *t*_50_ = 2.54, *P* = 0.01) and AP threshold (control: –51.52 ± 0.77 mV, patient: –48.63 ± 0.67 mV; *t*_49_ = 2.84, *P* = 0.007).

#### Abnormal post-translational processing and subcellular localization of *CSPG4*^*A131T*^ in OPCs

**Fig. 3:**
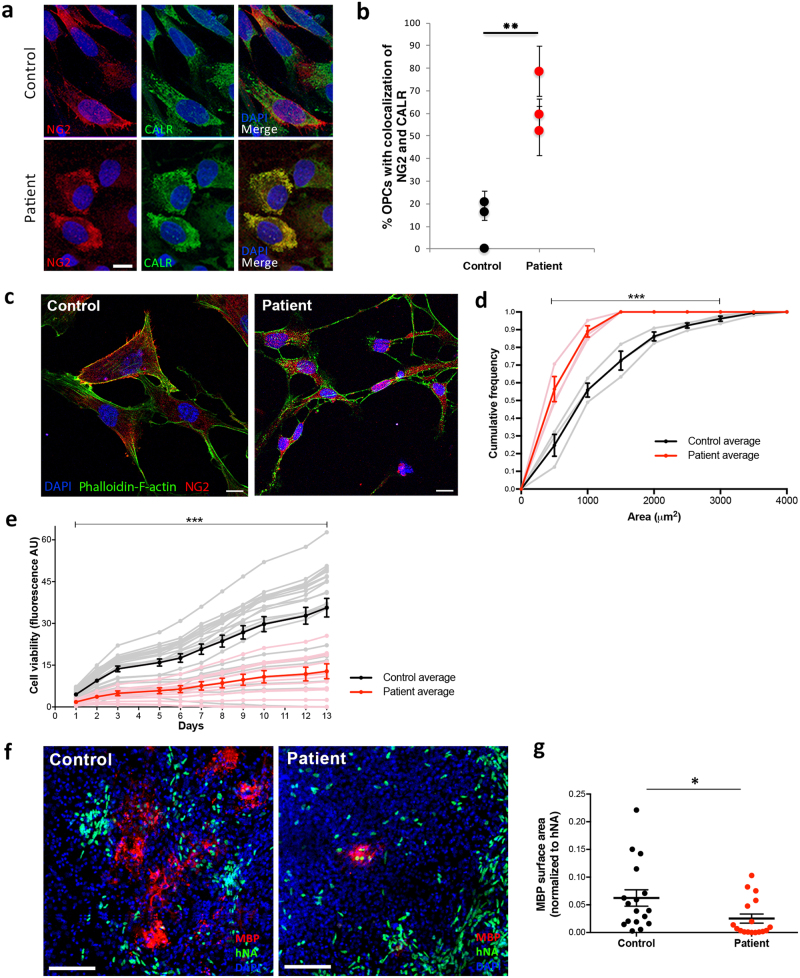
Aberrant NG2 subcellular localization, morphology, and viability of *CSPG4*^*A131T*^ patient OPCs. **a**, **b** Immunostaining for NG2 and calreticulin (CALR) reveals increased ER colocalization of NG2 in patient OPCs (scale bar = 10 µm). Quantification was performed in two independent experiments with three control and three patient OPC lines each (*t* = 5.08, *P* = 0.007). **c** OPCs derived from mutation carriers exhibit an abnormally small morphology (scale bars = 10 µm). **d** Cumulative distribution of OPC area (µm^2^; *n* = 361 control cells, *n* = 217 patient cells) demonstrates that OPCs derived from affected mutation carriers are significantly smaller than from their unaffected non-carrier siblings. Control and patient cell size distributions were compared by Kolmogorov–Smirnov test (*D* = 0.25, *P* = 3.0 × 10^−8^). Dark lines show group mean ± standard error. Gray and pink lines show the results from each of the individual control and patient subjects, respectively. **e** Fluorometric cell viability assay of OPCs derived from affected mutation carriers vs. their unaffected non-carrier siblings (two-way repeated measures ANOVA, *P* = 8.9 × 10^−7^). **f** Representative z-stacked images of organotypic cerebral cortex slices of homozygous shiverer mice transplanted with OPCs derived from affected mutation carriers or their unaffected non-carrier siblings. Human nuclear antigen (hNA), green; MBP, red (scale bar = 100 µm). **g** MBP surface area normalized to hNA^**+**^ cells per slice (*t* = 2.17, *P* = 0.038)

Given the highly abundant expression of NG2 in OPCs, widely referred to as NG2 cells, we next sought to investigate the influence of the *CSPG4*^*A131T*^ mutation on iPSC-derived OPCs. Directed differentiation of iPSCs to OPCs resulted in robust expression of the lineage-specific markers NG2, PDGFRα, Olig2, and SOX10 (Supplementary Fig. 3). We first examined the subcellular distribution of NG2, as the structural homology modeling of the *CSPG4*^*A131T*^ mutation suggested aberrant protein folding (Fig. 1c). Since NG2 is a transmembrane protein, it requires processing by the secretory pathway. Consistent with an impairment of protein processing, *CSPG4*^*A131T*^ patient-derived OPCs showed a highly abnormal subcellular localization of NG2 exemplified by an increase of co-localization with the endoplasmic reticulum marker calreticulin (*t* = 5.08, *P* = 0.007) (Fig. 3a, b).

In order to further characterize the alteration of NG2 subcellular localization, we performed surface biotinylation of *CSPG4*^*A131T*^ patient and non-carrier sibling control OPCs. NG2 is known to undergo extensive posttranslational modification [53, 54], including the addition of chondroitin sulfate moieties at Ser^999^ [55]. Consequently, NG2 appears as multiple bands by western blotting: a sharp band at 300 kDa corresponding to an unmodified form of NG2 which lacks chondroitin sulfate side chains, and a large polydisperse smear at >300 kDa corresponding to NG2 with chondroitin sulfate modification. Pre-incubation with chondroitinase ABC to enzymatically cleave the chondroitin sulfate side chains eliminated the >300 kDa polydisperse smear (modified NG2) and increased the 300 kDa band (unmodified NG2) (Supplementary Fig. 4a).

The total level of NG2 protein was similar between patient and control OPCs in whole-cell lysates (*t* = 0.51, *P* = 0.62) (Supplementary Figs. 4, 5). However, patient OPCs exhibited a significant decrease in the ratio of modified vs. unmodified NG2 compared to control OPCs. This finding was observed in whole-cell lysate (*t* = 2.88, *P* = 0.04), as well as independently in the intracellular (*t* = 3.50, *P* = 0.02) and surface protein fractions of OPCs (*t* = 3.31, *P* = 0.03) (Supplementary Fig. 4b, c). Taken together, these results demonstrate that the *CSPG4*^*A131T*^ mutation results in abnormal processing of NG2 protein.

#### Abnormal morphology of OPCs derived from *CSPG4*^*A131T*^ mutation carriers

In addition to the abnormal processing of NG2 protein, we also observed distinct morphological differences between OPCs derived from patients and controls (Fig. 3c). Patient-derived OPCs exhibited a size distribution that was strongly shifted toward smaller cells, a finding that was highly significant across all patient and control lines (Kolmogorov–Smirnov *D* = 0.25, *P* = 3.0 × 10^−8^; Fig. 3d). Overall, these results suggest that abnormal processing of NG2 influences the function of OPCs derived from *CSPG4*^*A131T*^ mutation carriers.

#### Patient OPCs have reduced viability and oligodendrogenesis

Abnormally high co-localization of mutant NG2 with calreticulin is consistent with retention of misfolded mutant NG2 in the endoplasmic reticulum. The extensive literature demonstrating impairments of cell viability resulting from misfolded proteins [56, 57] led us to hypothesize that the abnormal subcellular distribution of mutant NG2 might reduce the viability of OPCs. Therefore, we performed a longitudinal monitoring of cell viability using a quantitative fluorescence-based indicator based on the conversion of non-fluorescent resazurin to its fluorescent metabolite resorufin [28]. As evident throughout the entire 13-day time course of monitoring, OPCs derived from *CSPG4*^*A131T*^ mutation carriers exhibited a significant decrease of cell viability (two-way repeated measures ANOVA, *P* = 8.9 × 10^−7^; Fig. 3e).

OPCs are the exclusive precursor of oligodendrocytes [58]. Given the reduced viability of patient OPCs, we also investigated whether their maturation to oligodendrocytes might also be impacted. Therefore, we performed *ex vivo* co-culture of control and patient OPCs with organotypic slices of cerebral cortex from myelin-deficient Shiverer mice that carry a homozygous mutation of *Mbp*. The Shiverer myelination assay is a widely implemented method for assessing the myelination potential of OPCs, using culture conditions that promote OPCs differentiation to myelinating oligodendrocytes [30]. Consequently, MBP expression is entirely attributable to cells differentiated from the transplanted human OPCs, since Shiverer mice themselves lack Mbp. Consistent with their decreased viability, patient OPCs exhibited an impaired maturation to MBP-expressing oligodendrocytes (*t* = 2.17, *P* = 0.038; Fig. 3f, g).

#### Transfection of OPCs with mutant isoforms of NG2

**Fig. 4:**
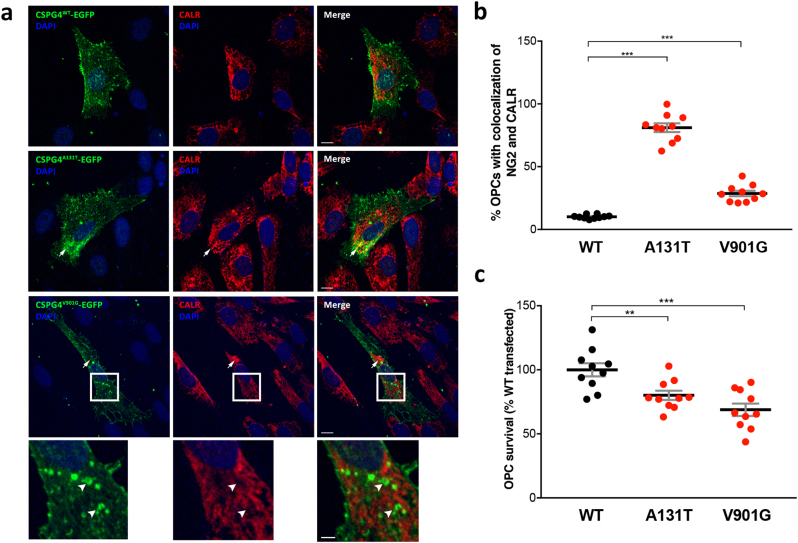
Transfection of *CSPG4*^*A131T*^ and *CSPG4*^*V901G*^ mutations in healthy non-carrier sibling OPCs. Control OPCs were transiently transfected with plasmids expressing WT or mutant CSPG4 isoforms fused to EGFP. **a** Confocal images of transfected cells confirm a normal extracellular membrane-localization of CSPG4^WT^-EGFP. In contrast, a high proportion of OPCs transfected with *CSPG4*^*A131T*^-EGFP exhibited colocalization with the endoplasmic reticulum marker calreticulin (CALR), reminiscent to patient OPCs with endogenous *CSPG4*^*A131*^. OPCs expressing *CSPG4*^*V901G*^-EGFP also revealed an increased proportion with CALR colocalization, but additionally demonstrated a distinctly abnormal targeting within putative intracellular vesicles that were negative for CALR (insets) (scale bars: main panels = 10 μm, insets = 3 μm). **b** Percentage of transfected OPCs with colocalization of CSPG4 isoforms with CALR (WT vs. A131T: *t* = 10.36, *P* = 5.2 × 10^−9^; WT vs. V901G: *t* = 6.20, *P* = 7.5 × 10^−6^). **c** Cell survival at 48 h after transfection of OPCs (WT vs. A131T: *t* = 3.11, *P* = 0.006; WT vs. V901G: *t* = 4.41, *P* = 3.4 × 10^−4^)

To further investigate the causality of the *CSPG4*^*A131T*^ and *CSPG4*^*V901G*^ mutations, we transfected either the wild-type (WT) or mutant isoforms into OPCs derived from healthy non-carrier siblings. Expression of *CSPG4*^*A131T*^ recapitulated the abnormal retention in the endoplasmic reticulum, similarly as observed in patient OPCs (one-way ANOVA, WT vs. A131T: *P* = 5.2 × 10^−9^; Fig. 4a, b). Moreover, OPCs expressing the *CSPG4*^*V901G*^ mutation also exhibited a significantly increased proportion of cells with co-localization to the endoplasmic reticulum compared to WT transfected OPCs (WT vs. V901G: *P* = 7.5 × 10^−6^), albeit lower than observed for the *CSPG4*^*A131T*^ mutation. Notably, however, expression of the *CSPG4*^*V901G*^ mutation resulted in a distinct accumulation within putative intracellular vesicles that was evident in both OPCs (Fig. 4a, inset) and U373 glioblastoma cells (Supplementary Fig. 6), but not observed with expression of the *CSPG4*^*A131T*^ mutation in either of these cell types. At present, although we have not yet succeeded in identifying the subcellular compartment to which these *CSPG4*^*V901G*^-expressing intracellular vesicles belong, lysosomes have been excluded based on the lack of significant co-localization with lysosomal-associated membrane protein 1 (Supplementary Fig. 6).

Lastly, we sought to evaluate the causality of *CSPG4* mutations for mediating the impaired cell viability observed for patient OPCs. Therefore, we performed transient transfections of either WT or mutant NG2 isoforms into OPCs derived from healthy non-carrier siblings and assessed cell survival after 48 h when plasmid expression was maximal. OPCs transfected with either *CSPG4*^*A131T*^ or *CSPG4*^*V901G*^ exhibited a significant decrease in survival compared to CSPG4^WT^ (one-way ANOVA, WT vs. A131T: *P* = 0.006; WT vs. V901G: *P* = 3.4 × 10^−4^; Fig. 4c). Notably, we also attempted the same experiment with patient OPCs; however, patient OPCs consistently died following the transfection procedure, regardless of whether the transfection was performed using WT or mutant NG2, or empty vector, a finding that independently confirms their distinct vulnerability.

### Impaired white matter microstructure in ***CSPG4***^*A131T*^ mutation carriers

**Fig. 5:**
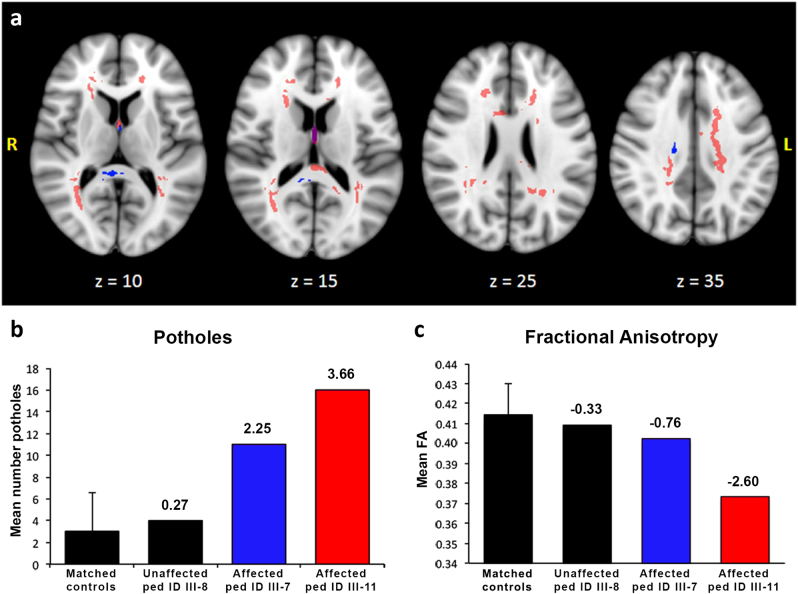
White matter abnormalities in *CSPG4*^*A131T*^ patients. **a** White matter potholes found in the two affected family members are shown in red and blue, respectively. Purple regions define a spatial overlap of potholes in both patients. The z-measures provide coordinates of the axial plane in MNI-space. **b** Mean number of potholes comparing the matched control population to the unaffected and two affected family members. **c** Whole-brain white matter FA comparing the matched general population subjects to unaffected and affected family members. Error bars in **b** and **c** reflect the standard deviation of the matched general population subjects. The number above each bar reflects the individual *z*-score compared to the matched general population group

Given the observed abnormalities of patient-derived OPCs, we reasoned that affected *CSPG4*^*A131T*^ mutation carriers might exhibit impairments of white matter integrity. Therefore, we performed brain MRI-based DTI in affected carrier and unaffected non-carrier siblings and compared them with 294 subjects from the general population Rotterdam Study cohort matched for age, gender, smoking behavior, and alcohol use (Fig. 5a). DTI images were analyzed for global and focal reductions in FA, the latter referred to as white matter potholes [59]. Affected carriers exhibited both a significantly higher number of white matter potholes (*P* = 2.2 × 10^−5^) and lower global FA (*P* = 8.2 × 10^−3^), compared to unaffected sibling and matched general population controls (potholes: 2.95 ± 3.52, 95% CI 2.55–3.35; global FA: 0.415 ± 0.015, 95% CI 0.413–0.417; Fig. 5b, c).

## Discussion

Our findings provide convergent genetic and functional neurobiological evidence that oligodendrocyte progenitor cell dysfunction might influence the pathophysiology of schizophrenia. We identified two different rare missense mutations in *CSPG4* exhibiting familial segregation with schizophrenia. The discovery family *CSPG4*^*A131T*^ mutation was associated with abnormal protein processing, OPC morphology, cellular viability, and oligodendrogenesis. The second mutation (*CSPG4*^*V901G*^), also located in a LamG domain, segregated with schizophrenia in two independent pedigrees and exhibited nominally significant enrichment in the Swedish Schizophrenia Exome Sequencing Study [8]. Moreover, induced expression of *CSPG4*^*A131T*^ and *CSPG4*^*V901G*^ isoforms confirmed the likely pathogenicity of these mutations for OPC function. Importantly, however, our findings leave still unaddressed the question of the extent to which there are distinct vs. shared pathophysiological mechanisms in schizophrenia attributable to common polygenic risk, CNVs, or other rare familial variants.

Our genetic findings highlight some of the challenges frequently encountered in the effort to identify pathogenic rare variants underlying common diseases such as schizophrenia. Although we identified three independent families exhibiting segregation of two different rare *CSPG4* missense variants with complete penetrance, none of these families was large enough on its own to achieve a genome-wide significant LOD score. Moreover, although the rare MAF of the *CSPG4*^*A131T*^ mutation precluded case/control validation in the Swedish Schizophrenia Exome Sequencing Study, two of the three *CSPG4*^*A131T*^ mutation carriers in the general population Rotterdam Study cohort had a clinically significant history of psychiatric illness that was unlikely due to chance. Furthermore, the *CSPG4*^*V901G*^ mutation was present in the Swedish cohort with a nominally significant enrichment in cases vs. controls. Additional follow-up studies in larger cohorts will be required to definitively evaluate the association between schizophrenia and rare *CSPG4* variants.

Genetic variation in *CSPG4* might confer a pleiotropic risk for mental illness since two of the *CSPG4*^*A131T*^ mutation carriers identified in the general population Rotterdam Study cohort had a clinically significant history of depression. Interestingly, a mouse model of OPC depletion was recently shown to exhibit depression-like behavior [60]. Such pleiotropic influences on mental health outcomes would be consistent with many of the previously identified genetic risk factors for schizophrenia [61, 62]. Furthermore, originally named as melanoma-associated chondroitin sulfate proteoglycan upon its cloning [63], NG2/CSPG4 has since been widely implicated in a wide variety of human cancers both as a diagnostic marker and a therapeutic target, including for glioblastoma and melanoma [64, 65].

The protein sequence surrounding the *CSPG4*^*A131T*^ mutation is conserved only among higher-order primates, suggesting recent evolutionary pressure. In contrast, the *CSPG4*^*V901G*^ variant is predicted to be a disease-causing mutation by PolyPhen2 [66]. In non-primate vertebrates, the reference amino acid at position 131 is threonine, corresponding to the patient mutation *CSPG4*^*A131T*^ and contributing to a benign PolyPhen score. Interestingly, this is analogous to the human *SNCA*^*A53T*^ mutation, one of the most well-established mutations for autosomal dominant Parkinson’s Disease [67]. In mice, the reference amino acid at *SCNA* position 53 is a threonine. But notably, although transgenic expression in mice of the reference human *SCNA* sequence is benign, introduction of the human *SNCA*^*A53T*^ mutation is highly pathogenic [67].

To investigate the cellular pathophysiology resulting from mutation of *CSPG4*, we derived iPSCs from affected and unaffected siblings of the discovery family. Detailed electrophysiological analysis of patient-derived neurons revealed an increase in input resistance and depolarized shift in AP threshold. Multiple previous studies have demonstrated primary functional neuronal mechanisms in human iPSC models of schizophrenia [3, 8, 11–13, 68]; however, the changes we observed are distinct from those previously identified. Therefore, future studies will be required to determine the extent to which these differences may have pathophysiological relevance, and potentially reflect an increased burden of common polygenic risk as recently observed in familial schizophrenia [69]. Importantly, however, our limited findings in neurons compared to OPCs cannot be considered to imply that neuronal dysfunction is not central to the pathophysiology of schizophrenia. Rather, these data suggest that although the symptoms of schizophrenia are ultimately manifest from neuronal dysfunction, the primary pathophysiological mechanism could be mediated by direct neuronal impairments and/or indirectly through non-neuronal cell types including OPCs, depending upon the nature of the etiological factors driving disease risk in a given individual.

OPCs receive extensive GABAergic input from surrounding neurons, which regulate their differentiation to myelinating oligodendrocytes [70–73]. However, it has remained less well understood whether OPCs directly modulate neuronal function independent of myelination. Notably, two recent studies have suggested novel candidate mechanisms by which OPC dysfunction might directly regulate neuronal function, including activity-dependent ecto-domain cleavage of NG2 [54] and local buffering of extracellular potassium [74].

Regarding the identified *CSPG4* mutations demonstrating familial segregation with schizophrenia, alterations in myelination are a parsimonious candidate mechanism given the convergent findings of genetic mutations in the OPC marker protein *CSPG4*, functional impairments of iPSC-derived OPCs, and *in vivo* DTI-based structural brain imaging. Moreover, the abnormal subcellular localization of NG2 in OPCs expressing the *CSPG4*^*A131T*^ or *CSPG4*^*V901G*^ isoforms is consistent with the high susceptibility of cells of the oligodendrocyte lineage to disruptions in the secretory pathway, as their maturation requires a substantial upregulation of membrane protein expression [75]. Interestingly, earlier work has shown a precedent for altered subcellular localization of NG2, in which mutation of Ser^999^ results in an abrogation of chondroitin sulfate side chain modification and altered subcellular localization [55].

Taken together, these findings are highly consistent with the growing body of evidence implicating white matter integrity in schizophrenia neuropathology [59, 76–79]. Indeed, recent findings demonstrating myelination of parvalbumin-positive GABAergic interneurons—arguably the most well-established neuronal cell type implicated in the pathophysiology of schizophrenia [80, 81]—raises the intriguing possibility that schizophrenia might result from neurodevelopmental alterations of PV interneuron myelination [82].

In summary, our findings support the validity of family-based genetics and iPSC modeling to unravel the underlying mechanisms of complex, heterogeneous psychiatric diseases, and provide evidence in support of oligodendrocyte precursor cell dysfunction as a novel candidate mechanism of schizophrenia.

## Electronic supplementary material

Supplementary Information

Supplementary Figure 1

Supplementary Figure 2

Supplementary Figure 3

Supplementary Figure 4

Supplementary Figure 5

Supplementary Figure 6

Supplementary Figure 7

Supplementary Table 1

Supplementary Table 2

Supplementary Table 3

Supplementary Table 4

Supplementary Table 5
