# Supplementary material for: Candidate CSPG4 mutations and induced pluripotent stem cell modeling implicate oligodendrocyte progenitor cell dysfunction in familial schizophrenia
Source: Mol Psychiatry. 2018 Jan 4;24(5):757–71. doi: 10.1038/s41380-017-0004-2 (PMC6755981; doi:10.1038/s41380-017-0004-2)
Supplement: Supplementary file 1 — Supplementary Information [file 41380_2017_4_MOESM1_ESM.docx]

**SUPPLEMENTARY FIGURE LEGENDS**

**Supplementary Figure 1. Additional rare coding variants in *CSPG4*.**

**(a)** Pedigrees with *CSPG4^R641W^*, *CSPG4^R1179W^*, and *CSPG4^R1980H^* variants. Symbols: filled, schizophrenia; open, unaffected; heterozygous carriers of the *CSPG4* c.1921C>T p.R641W, c.3535 C>T p.R1179W, and c.5939 G>A p.R1980H. **(b)** Representative sequencing results for heterozygous carriers of the corresponding *CSPG4* mutations in panel A.

**Supplementary Figure 2. Characterization of iPSC clones and NPCs.**

**(a)** RT-PCR of pluripotency genes normalized to GAPDH expression levels confirmed down-regulation of exogenous reprogramming genes and up-regulation of endogenous stem cell genes, similar to H9 human embryonic stem cells. **(b)** iPSC colonies showed uniform staining for pluripotency markers Nanog, OCT4 and TRA1-81. **(c)** EB differentiation confirmed that iPSCs were capable of generating representative cell types of all 3 embryonic layers – endoderm (AFP), mesoderm (Vimentin) and ectoderm (GFAP) (scale bar = 20 μm). **(d)** Karyotyping was performed on all iPSC clones to confirm genomic integrity following reprogramming. Shown is a representative karyogram from the iPSC line of subject III-11. **(e)** NPCs were positive for SOX2, Nestin, Vimentin and FOXG1, confirming their forebrain specification (scale bar = 10 μm).

**Supplementary Figure 3. Characterization of OPCs.**

NG2**^+^** cells co-expressed the OPC markers PDGFRα (**a**), Olig2 and SOX10 (**b**) (scale bars = 15 μm).

**Supplementary Figure 4. Aberrant post-translational chondroitin sulphate modification in CSPG4^A131T^ patient-derived OPCs. (a)** Chondroitin sulphate side chains are specifically cleaved by chondroitinase treatment. **(b)** Representative western blot of biotinylation assay samples of control and patient OPCs +/- chondroitinase ABC treatment (intra = intracellular fraction, surf = surface fraction, total = whole cell lysate fraction). **(c)** Quantification of the proportion of chondroitin sulphate-modified NG2 protein. The proportion of modified to unmodified NG2 in OPC extracts was determined by normalizing the integrated density of modified NG2 (>300 kDa) to the total integrated density of the modified plus unmodified NG2 bands. Experiments using control (n=9) and patient (n=8) OPCs are shown as aggregated data per individual subject (3 control and 3 patient siblings). Error bars in all panels reflect standard errors of the mean. Unpaired two-tailed Student’s t-test revealed significantly less modified NG2 protein in whole cell lysate (t=2.88, P=0.04), intracellular (t=3.50, P=0.02) and surface fractions (t=3.31, P=0.03). **(d)** Control experiment showing that following chondroitinase ABC treatment, the abundance of 300 kDa (unmodified) NG2 in the whole cell lysate fraction is similar between patient and control OPCs (n=6 control and 6 patient clones, t=0.51, *P*=0.62). **(e)** In the absence of biotin (left panel), NG2 protein is undetectable in the Neutravidin-purified surface fraction. Furthermore, the absence of detectable actin in the surface protein samples confirms the high specificity of the biotin purification.

**Supplementary Figure 5. Raw LI-COR western blot images.**

Full western blots for NG2 protein (upper) and re-blotting for actin (below). **(a)** Full blot corresponding to Figure 3D (lower half cut vertically for processing). **(b)** Full blot corresponding to Supplementary Figure 4b.

**Supplementary Figure 6. CSPG4^V901G^ exhibits abnormal protein localization in U373 human glioma cells.** Transfection of CSPG4^V901G^-EGFP expression in human U373 glioma cells that lack endogenous CSPG4 demonstrates abnormal localization in vesicular structures that do not colocalize with lysosome marker LAMP1.

**Supplementary Figure 7. *CSPG4P13* pseudogene sequencing controls.**

**(a)** Partial alignment of *CSPG4* Taqman amplicon (hg19 chromosome 15: 75982916-75983072) with homologous *CSPG4P13* pseudogene sequence (hg19 chromosome 15: 78188526-78188682). Filled arrow indicates the position of the *CSPG4* c.391G>A variant. Open arrow indicates the differential nucleotide distinguishing the flanking *CSPG4* sequence from *CSPG4P13*. **(b)** Sanger traces of an individual carrying the *CSPG4* c.391G>A variant while homozygous reference for *CSPG4P13*. **(c)** Sanger traces of a sample called falsely positive by Taqman genotyping due to a homologous *CSPG4P13* polymorphism (genomic position, hg19 chromosome 15: 78188583) while being homozygous reference for *CSPG4* c.391G.

**SUPPLEMENTARY METHODS**

**Recruitment of family with schizophrenia and obtaining patient material**

A Dutch white Caucasian non-consanguineous family with a high incidence of schizophrenia was ascertained. Written informed consent was obtained from all participating subjects. This study was approved by the medical ethical committee of the Erasmus University Medical Centre (Rotterdam, The Netherlands). Participating family members were screened for current or past psychiatric symptoms using the Structural Clinical Interview for DSM-IV (SCID-1)^1^. Medical screening revealed no evidence of somatic comorbidities, dysmorphologies, or neurological symptoms. All individuals with schizophrenia were documented to be of average intelligence at the time of their initial diagnosis. DNA isolation from venous blood samples and skin biopsies were performed using standard procedures. Two family members (II-2 and III-9) passed away since the start of the study.

For skin biopsies, a small area of skin of the medial aspect of the upper arm was anesthetised with an EMLA patch (AstraZeneca) for one hour, after which the skin was disinfected with ethanol and the biopsy was obtained through all skin layers with a standard 3mm biopsy punch. The tissue was collected in Dulbecco's Modified Eagle Medium (DMEM) (Gibco-Invitrogen) without additives and transferred to culture within 24 hours. Primary human fibroblasts were cultured in DMEM containing 10% fetal calf serum and 1% penicillin/streptomycin (P/S).

**Genetic analysis**

Genomic DNA was isolated using standard methodology from 14 family members, including 13 by whole blood samples and one subject (pedigree ID II-1) using paraffin-embedded tissue (**Figure 1a**). Linkage and Copy Number Analysis was performed with Illumina HumanCytoSNP-12v2 chip arrays (294,975 markers). Linkage analysis was conducted exclusively for the purpose of identifying a genome-wide set of candidate chromosomal regions shared by all affected family members (defined as those regions with a LOD score > 0). Linkage was performed using Allegro and implemented in the EasyLinkage version 5.08 interface ^2^ with one marker every 0.5 cM using a co-dominant allele frequency algorithm and dominant scoring function. Unaffected family members were considered as having an unknown affection status in the linkage model, providing no *a priori* constraints on the rate of incomplete penetrance. Copy number analysis was performed using NEXUS discovery edition, version 6 (BioDiscovery, El Segundo, CA), which did not indicate the presence of copy number variants segregating with the phenotype. The strongest evidence of linkage was observed on chromosomes 2, 11, 14, 15, and 16, involving a total of 294.34 Mb of genomic loci.

Whole exome sequencing was performed on three individuals of the discovery family (pedigree IDs: II-2, III-5 and III-9). Sequencing was performed twice for all three samples: initially at 40x, and subsequently at 90x coverage. Exome sequencing was performed using in-solution capturing (Agilent SureSelect V2 and V4 Human 50 Mb kit respectively, Agilent Technologies) and paired-end sequencing with Illumina Hi-Seq 2000 sequencers. Reads were aligned to the human reference genome version 19 using Burrows-Wheeler Aligner. SNPs and indels were called using the Genome Analysis Toolkit (GATK).

The heterozygous variants were filtered based on the following criteria: a) present within the candidate genomic regions shared among all affected family members, b) predicted to affect protein coding (missense, nonsense, frameshift, splice site), c) called in at least one of the affected individuals [III-5 and III-9], d) absent from the unaffected mother [II-2], e) absent from dbSNP129, and f) with a minor allele frequency (MAF) of < 0.001 in in the Exome Aggregation Consortium (ExAc) browser (European non-Finnish)^3^, Exome Variant Server (EVS6500, NHLBI Exome Sequencing Project)^4^, 1000 Genomes^5^, and Genome of the Netherlands^6^ cohorts.

Genotyping of *CSPG4* c.391G>A was performed in a cohort of Dutch subjects with 1219 schizophrenia cases and 10,611 controls. Genotyping of 763 schizophrenia cases and 386 healthy control subjects were performed using a custom Illumina Infinum Human Exome Beadchip array (Genetic Risk and Outcome of Psychosis [GROUP] / Utrecht cohort study^7^). TaqMan genotyping was performed on an additional 456 locally-collected schizophrenia cases and 10,225 population-based controls filtered for identity by descent ($\hat{\pi}$ < 0.25) (Rotterdam Study^8^). All samples with ALT calls by exome array or TaqMan genotyping were validated by Sanger sequencing, particularly given the existence of the *CSPG4P13* pseudogene with high homology to the region surrounding *CSPG4* c.391G>A (**Supplementary Figure 7**).

Taqman genotyping of *CSPG4* c.391G>A was performed with the following primers and probes: F_GGCTGGTTCCCCTCAGGTA, R_GTGGTGCTGACTGTCGTAGAG,

VIC_TTTCTGAACGCCTCCTC, FAM_TTTCTGAACACCTCCTC.

The following primer sets were used for Sanger sequencing of *CSPG4* c.391G>A: F_TCTGGGGCCCCAAGTGTGG and R_AGAGTGGGGCCCAGAGAAGC, with an internal forward primer for sequencing: Fseq_GGGCCAGGAGGAGCTGAGG. A second primer set was used for confirmation: F2_CCACTCCCCATCTCTCTTCAGG and R2_CAGGGCCACATCATCACTGG.

*CSPG4P13* pseudogene-specific amplification was performed using the following primers: F_CTCTGGGGCACCAAGAGTGG and R_AGAGTGGGGCCCAGAGAAGC.

**Structural homology modeling**

Homology modeling of the first Laminin G domain (amino acids 29-176) was performed independently using both the Protein Homology/analogY Recognition Engine – Version 2.0 (Phyre2)^9^ and Iterative Threading ASSEmbly Refinement (I-TASSER)^10^ protein structure prediction servers. All 148 residues (100%) of the reference and mutant sequences were modeled at >99% confidence by Phyre2 using intensive mode. For I-TASSER, the confidence (C-score) was 0.81 and topological similarity (TM-score) was 0.82+/- 0.08, for both models. Reference and mutant models were structurally aligned with PyMol (http://www.pymol.org).

**Generation and characterization of iPSCs**

Reprogramming of human primary skin fibroblasts was performed as previously described^11^. Briefly, fibroblasts were infected with a multicistronic SIN lentiviral vector containing an SFFV promoter, encoding OCT4, SOX2, KLF4 and MYC, as well as dTomato to visualize reprogramming. Emerging iPSC colonies were cultured on γ-irradiated mouse embryonic feeder (MEF) cells. All iPSC lines and their derivatives used in this study were screened monthly for mycoplasma.

Characterization of iPSC clones was performed by RT-PCR (**Supplementary Figure 2a**), immunostaining for un-differentiated human ES markers (**Supplementary Figure 2b**) and markers of three embryonal germ layers on embryoid bodies (EBs) differentiated *in vitro* (**Supplementary Figure 2c**). Total RNA of iPSCs was isolated for RT-PCR using standard protocols (primers listed in **Supplementary Table 5)**. For EB differentiation, iPSC colonies were dissociated by collagenase IV treatment and transferred to ultra-low attachment 6-well plates (Corning). Floating EBs were cultured in iPSC medium without bFGF for a minimum of 6 days with supplemented SB431542 (Tocris Bioscience) for ectoderm conditions only. EBs designated for endoderm differentiation were transferred to gelatin-coated 12-well plates containing the following medium: RPMI 1640 (Gibco-Invitrogen), supplemented with 20% FBS, 1% P/S, 1% glutamine and 0.4mM alpha-thioglycerol. Mesoderm differentiation from the EBs was induced in gelatin-coated 12-well-plates with DMEM medium (low glucose) supplemented with 15% fetal bovine serum, 1% P/S, 1% glutamine and 1% MEM-non-essential amino acids. Ectoderm differentiation was induced in Matrigel (BD)-coated plates with the following medium: neurobasal medium (Gibco-Invitrogen) and DMEM/F12 (v/v 50/50) supplemented with 1% P/S, 1% glutamine, 1% MEM-non-essential amino acids, 0.02% BSA (Gibco-Invitrogen), 0,5% N2 (Gibco-Invitrogen) and 1% B27 (Gibco-Invitrogen). After two weeks in culture, cells were fixed with 4% formalin for immunolabeling.

iPSCs were cultured in standard human embryonic stem (hES) cell culture medium containing DMEM/F12 (Gibco-Invitrogen) supplemented with 20% knock-out serum replacement (Gibco-Invitrogen), 2mM L-glutamine (Gibco-Invitrogen), 1% P/S (Gibco-Invitrogen), 1% MEM-non-essential amino acids (PAA Laboratories GmbH), 0.1mM β-mercapto-ethanol, and 10 ng/ml bFGF (Gibco-Invitrogen). Medium was replenished daily and colonies were passaged weekly using collagenase IV (1 mg/ml, Gibco-Invitrogen) with 10µM ROCK inhibitor (Y-27632, Sigma).

**Karyotype analysis**

iPSCs were dissociated to single-cell suspension using TrypLE Express (Gibco-Invitrogen) and plated feeder-free in mTeSR1 medium on three Matrigel-coated wells of a 6-well plate in the presence of 10µM ROCK inhibitor. The next day, cells were harvested using TrypLE Express, treated with colcemid (200 ng/ml) and hypotonic solution, and fixed using Carnoy’s Fixative. At least 20 metaphases were analysed for each clone, for which the chromosome count was considered normal if more than 70% of cells analysed had 46 chromosomes. More detailed karyotypic analysis with RBA and QFQ band analysis was performed for one individual patient clone (individual III-11) to further exclude segregating cytogenetic abnormalities (**Supplementary Figure 2d**).

**Neuronal differentiation**

iPSC colonies were dissociated from MEFs with collagenase IV and transferred to non-adherent plates in hES cell medium on a shaker in an incubator at 37ºC/5% CO_2_. After two days, EBs were changed to neural induction medium [DMEM/F12, 1% N2-supplement (Gibco-Invitrogen), 2 µg/ml heparin (Sigma), 1% P/S] and cultured for another four days in suspension. EBs were gently dissociated and plated onto laminin-coated dishes in neural induction medium. Cells were dissociated with collagenase IV after 8 days and plated onto laminin-coated dishes in NPC medium [DMEM/F12, 1% N2, 2% B27-RA, 1 µg/ml laminin (Sigma) and 20 ng/ml FGF_2_ (Millipore), 1% P/S]. After one week, NPCs were dissociated with collagenase IV, re-plated, and passaged 1:4 weekly. For neural differentiation, passage 5 NPCs were plated on coverslips coated with 100 µg/ml poly-L-ornithine (Sigma) and 50 µg/ml laminin in neural differentiation medium consisting of Neurobasal medium supplemented with 1% MEM-non-essential amino acids, 1% N2 supplement, 2% B27-RA supplement, 20 ng/ml BDNF (ProSpecBio), 20 ng/ml GDNF (ProSpecBio), 1 μM db-cAMP (Gibco-Invitrogen), 200 μM ascorbic acid (Gibco-Invitrogen), 2 μg/ml laminin and 1% P/S.

**Electrophysiological recordings**

After 8-10 weeks of neuronal differentiation, culture slides were transferred to the recording chamber following a thirty-minute serial partial exchange of cell culture medium with artificial cerebrospinal fluid (ACSF) containing the following (in mM): 110 NaCl, 2.5 KCl, 2 CaCl_2_, 2 MgCl_2_, 1 NaH_2_PO_4_, 25 NaHCO_3_, 10 glucose, 0.2 ascorbate (pH 7.4). In the recording chamber, slides were continuously perfused with ACSF at 1.5-2 mL/min, saturated with 95% O_2_/5% CO_2_ and maintained at 20-22°C.

Whole-cell patch-clamp recordings were performed under infrared differential interference contrast visual guidance using an upright microscope (Zeiss) with borosilicate glass recording micropipettes (3-6 MΩ) filled with the following medium (in mM): 130 K-gluconate, 11 KCl, 10 HEPES, 5 NaCl, 0.1 EGTA, 1 MgCl_2_, 2 Mg-ATP, 0.3 Na-GTP, 5 phosphocreatine (pH 7.4). Data were acquired at 10 kHz using an Axon Multiclamp 700B amplifier (Molecular Devices), filtered at 3 kHz, and analyzed using pClamp 10.1 (Molecular Devices). Whole-cell capacitance and series resistance were compensated, and voltage was adjusted for liquid junction potential.

Current-clamp recordings were performed at a holding potential of ‑60 mV. Passive membrane properties were analysed using a series of hyperpolarizing and depolarizing square wave currents (500 msec duration, 1 sec interstimulus interval) in 5 pA steps, ranging from ‑20 to +30  pA. AP amplitude, rise time, decay time and half width were measured for the first evoked AP resulted by a depolarizing step, from the threshold to the peak, for which the threshold was defined by the moment at which the second derivative of the voltage exceeded the baseline. Spontaneous APs were recorded at resting membrane potential. Voltage-clamp recordings were performed at a holding potential of -90 mV.

**Oligodendrocyte lineage differentiation**

iPSC-derived NPCs were differentiated to OPCs according to Monaco *et al*^12^, with modifications. NPCs were plated on laminin-coated 10 cm plates in NPC medium consisting of DMEM/F12, 1% N2, 2% B27-RA, 1 µg/ml laminin, 1% P/S, 25 ng/ml basic Fibroblast Growth Factor (bFGF) and 20 ng/ml Epidermal Growth Factor (EGF).

When NPCs reached 90% confluence, NPC medium was changed to OPC differentiation medium for three weeks: DMEM/HAMS F12, 1% N2, 1% BSA, 1% L-Glutamine, 1% P/S, 20 ng/ml bFGF, 10 ng/ml platelet derived growth factor (PDGF-AA, ProspecBio), 2ng/ml Sonic hedgehog (Shh), 2 ng/ml neurotrophic factor 3 (NT-3) and 3 nM triiodothyronine (T3). OPC medium was changed every other day, and cells were passaged weekly at 1:4.

**Surface Biotinylation**

Biotinylation of cell surface proteins was adapted from Huang *et al*^13^, with modifications. First, the OPCs were washed twice in cold PBS/CaCL2/MgCl2 (2.5 mM CaCl2, 1 mM MgCl2, pH 7.4). Then, the OPCs were incubated for 15 minutes on ice with or without 0.3mg/ml of freshly dissolved Sulfo-NHS-SS-Biotin (Thermo Scientific) in PBS/CaCL2/MgCl2. Next, the OPCs were washed three times on ice in cold biotin quenching solution consisting of 50 mM glycine in PBS/CaCl2/MgCl2. Subsequently, the OPCs were lysed by incubation for 10 minutes on ice with lysis buffer, pH7.4, consisting of 5mM EDTA, 5mM EGTA, 1% Phosphatase inhibitor cocktail 2 and 3 (Sigma), 1% PIC (Sigma), and 1% Triton X in PBS. At least 3 wells were pooled from each cell line at each experimental condition, to minimize any plating or growing variability in the cell culture. A portion of the lysate was removed and labeled as the total fraction (whole cell lysate). The remaining lysate was then centrifuged (13200 rpm at 4°C for 15 minutes) to remove insoluble proteins. The supernatants were incubated overnight at 4°C with PBS-washed and lysis buffer-equilibrated Pierce™ NeutrAvidin™ Agarose beads (Life Technologies). Next, the mixture was spun down at 2000 rpm, separating the supernatant termed the intracellular fraction from the surface protein fraction attached to the beads. Subsequently, the beads were washed four times by rotating with lysis buffer and after the final wash the beads were dried completely and taken up in 20µl of lysis buffer, labeled as the surface fraction.

**Sodium dodecyl sulfate (SDS)-PAGE and Western Blotting:**

First, 25% XT Sample Buffer (Biorad) and 10% 100mM dithiothreitol (DTT) were added to equal volumes of cell extract followed by heating to 65°C for 15 minutes. Then, the samples were electrophoresed through 4%-12% Criterion™ XT Bis-Tris gradient gels (Biorad) in XT-Mops buffer (Biorad). Proteins were transferred overnight at 4°C on 0.45µm pore nitrocellulose membrane (Biorad) in a Tris-Glycine buffer consisting of 10% Tris/Glycine buffer (Biorad) and 20% anhydrous methanol in distilled water. Membranes were blocked for 2 hours while shaking at room temperature in TBST buffer containing 4% blotting grade blocker (Biorad). Then, the membranes were incubated with a monoclonal antibody generated from hybridoma B5 cells raised against purified intact NG2 protein from melanoma cells (generous gift from W.B. Stallcup) and mouse anti-actin (Sigma) in TBST buffer containing 1% Tween and 2% milk for 48 hours at 4°C followed by washing with TBST buffer. Finally, the blots were incubated with IRDye® secondary antibodies (LI-COR) against the primary antibody species for two hours at room temperature. The blots were washed twice in TBST and TBS followed by one wash in water, subsequently the antibody fluorescence was visualized using an ODYSSEY® CLx scanner (LI-COR). Quantification of NG2 western blot bands was performed using ImageJ software (http://imagej.nih.gov/ij/).

***Shiverer* co-culture assay**

Experiments were approved by the Dutch Ethical Committee and in accordance with the Institutional Animal Care and Use Committee (IACUC) guidelines. *Shiverer* mice (*C3Fe.SWV-Mbp^shi^*, Jackson Laboratory) were backcrossed >10 generations into *C57BL/6J*. Organotypic cortex slices were established from homozygous shiverer (*Mbp^shi/shi^*) mice at P3-P5 according to Stoppini *et al^14^*, with modifications. Briefly, the brain rapidly removed and transferred to ice cold Gey's Balanced Salt Solution (Sigma) containing 5.4 mg/ml glucose and 1% P/S. After dividing the brain sagittally into two hemispheres, 300 μm fronto-parietal coronal slices were obtained using a tissue chopper (McIlwain). Slices were cultured on an air-fluid interface at 37°C with 5% CO, using culture plate inserts (Millipore: 0.4 μm pore size; 30 mm diameter; 3 cultures per insert) in 1.0 mL culture medium containing 50% MEMα, 25% HBSS, 25% horse serum, 6.5 mg/mL glucose, 2 mM glutamine, 1% N2 supplement and 1% P/S, supplemented with PDGF-AA. For each *Mbp^shi/shi^* mouse, adjacent slices were randomly assigned for transplantation with either patient or control OPCs, thereby providing comparative matched pairs. Each slice was transplanted with 1 x 10^4^ iPSC-derived OPCs in 1.0 μL PBS containing 0.1% FastGreen (Gibco-Invitrogen) using a Picospritzer. OPCs were allowed to differentiate into myelinating oligodendrocytes for 10 days, following the procedure described in Najm *et al*^15^.

**Immunocytochemistry and confocal imaging**

Cell cultures and organotypic slices were fixed using 4% formalin in PBS. Primary antibodies were incubated overnight at 4°C in labeling buffer containing 0.05 M Tris, 0.9% NaCl, 0.25% gelatin, and 0.5% Triton-X-100 (pH 7.4). The following primary antibodies were used: SOX2 (AB5603), Nestin (MAB5326), MAP2 (AB5622), NeuN (MAB377), GFAP (AB5804), human Nuclear Antigen (hNA, MAB1281) [Millipore], FOXG1 (45-053) [ProSci], NG2-EC [kindly provided by W.B. Stallcup], Olig2 (AB42453), MBP (AB7349) [Abcam], Vimentin (SC6260) [Santa Cruz Biotechnology], AFP (MAB1368), SOX10 (AF2864) [R&D Systems], TRA-1-81 (560072), Nanog (560482) [Beckton Dickinson], OCT4 (AB19857) [Abcam], Calreticulin (PA3-900) [Fisher Scientific]. The following secondary antibodies were used: Alexa-488, Alexa-546, Alexa-555 and Cy3 antibodies [Jackson ImmunoResearch]. Samples were imbedded in Mowiol 4-88 (Fluka) after which confocal imaging was performed with a Zeiss LSM700 confocal microscope (Apochromatic 40x objective, 1.3 NA, oil immersion) using ZEN software (Zeiss, Germany).

Experimenters were blinded to group allocation when performing experiments, during data collection, and when assessing outcomes. OPC area (µm^2^) was quantified on maximum projection of z-stack images covering the full depth of each measured cell using a manually drawn surface contour in ImageJ. Quantification of human nuclei (hNA) and myelin (MBP) content of organotypic cortex slices was performed using Image J. Maximum intensity projections of tiled z-stacks covering the full dimensions of each slice with a constant interval of 1.0 μm were converted to individual 8-bit threshold images. Human nuclei (hNA) images were further segmented using a binary watershed filter. The number and surface area of labelled cells were quantified using the particle analysis algorithm. Cell viability was measured using resazurin (AlamarBlue, Thermo Fisher Scientific) in 96-well plates. Fluorimetric data were collected using a plate reader at a wavelength of 570 nm excitation and 585 nm peak emission. The data were normalised for the number of living cells using calcein AM (Thermo Fisher Scientific) at 488 nm excitation and 517 nm peak emission and quantified in adjacent wells seeded concurrently with equal numbers (2000 cells/well).

**Plasmid Transfections**

Control OPCs and U373 glioma cells were transfected with cDNA constructs to express fusion proteins of CSPG4 and its mutant variants with C-terminal EGFP. *CSPG4* cDNA sequence (*CSPG4^WT^*) was cloned into the Gateway vector pcDNA-DEST47 (pcDNA-DEST47-*CSPG4^WT^*) and converted to *CSPG4^A131T^* by site-directed mutagenesis. The corresponding plasmid containing the *CSPG4^V901G^* sequence was obtained by sub-cloning a custom-ordered V901G-containing fragment (GeneArt, Thermo Fisher Scientific) into the pcDNA-DEST47-*CSPG4^WT^* vector. All three plasmids were confirmed by sequencing. OPCs were transfected with *CSPG4^WT^-EGFP*, *CSPG4^A131T^-EGFP*, or *CSPG4^V901G^-EGFP* using X‑TremeGENE transfection reagent (Sigma-Aldrich) with 0.1 μg DNA per 96-well, or 1.0 μg DNA per 12-well. Cells were fixed 48 hours after transfection.

**Magnetic Resonance Imaging (MRI)**

Two patients and two control siblings of the discovery family provided additional written informed consent for MRI scanning. One of the unaffected siblings was excluded due to a history of systemic chemotherapy known to influence white matter integrity^16^. Population controls (n=294) were selected from the Rotterdam Study based on matching for age, gender, smoking behaviour, and alcohol use. All subjects, including population controls and family members, were imaged using the identical MRI scanner and acquisition protocol.

MRI images were obtained using a 1.5 Tesla General Electric scanner (GE Healthcare, Milwaukee, Wisconsin, USA, software version 11X), with a bilateral phased-array head coil. A full description of the imaging protocol has been described previously^17^. Diffusion-weighted imaging was performed using echo planar imaging (EPI) sequences collected in 25 directions with a b value of 1,000 s/mm^2^ and three b = 0 images. Additional sequence parameters were TR = 8,000 ms, TE = 74.6 ms, bandwidth 14.71 kHz, flip angle = 13, acceleration of 2, and a voxel resolution of 3.3 x 2.2 x 3.5 mm^3^.

Pre-processing of the diffusion-weighted images was performed using FSL^18^. After conversion from dicom to nifti format, individual images were eddy-current corrected using FMRIB’s eddy_correct^18^ followed by skull stripping using BET^19^. Fractional anisotropy (FA) images were created by fitting a tensor model to the diffusion data using FMRIB's Diffusion Toolbox (FDT)^18^. All subjects' FA data were then aligned into common space using the nonlinear registration tool FNIRT^20^, which employs a b-spline representation of the registration warp field^21^. Whole brain mean FA was calculated by masking each image using the Johns Hopkins University White Matter Atlas (JHU WMA)^22^ and determining the mean intensity of FA within the mask.

An in-house MATLAB (Mathworks, Natick, MA) algorithm was used to quantify the number and spatial characteristics of white matter ‘potholes’ along the major white matter tracts^23^. The input to the algorithm was the set of FA images that had undergone non-linear registration into MNI space using TBSS^24^. No spatial filtering was applied to the images. The first step was to generate a voxel-by-voxel mean and standard deviation (SD) image of the 294 matched population controls. These group and SD images were then used to individually create a voxel-wide z-image for all of the subjects, including the three family members. This resulted in individual z-images with each voxel based on the mean and SD of the matched population controls. To ensure the search involved only white matter regions, each image was masked with the cortical areas defined by the Johns Hopkins University white matter atlas^22^.

The individual z-FA images were then used to search for contiguous voxels of white matter below a threshold of z < -2^23,25^. Clusters were determined by thresholding each image and labeling contiguous voxels in three-dimensional space that fell below the defined threshold. Only clusters greater than 250 voxels were used in the analyses.

**Statistical Analysis**

Significance of observations was established for genetic case-control analyses using the Fisher’s exact test. Brain imaging results in family members were evaluated using *z*-scores based on the matched population control cohort distribution. For functional studies, statistical comparisons were performed using two-tailed t-test, analysis of variance (ANOVA), or Kolmogorov-Smirnov test, as indicated. Data are expressed as mean ± S.E.M., unless otherwise specified. The threshold for significance was set at *P<*0.05 for all statistical comparisons.

**SUPPLEMENTARY REFERENCES**

1 First M, Gibbon M, Spitzer R. Structured Clinical Interview for DSM-IV Axis II Personality Disorders (SCID-II). *Am Psychiatr Press Inc* 1997.

2 Hoffmann K, Lindner TH. easyLINKAGE-Plus - Automated linkage analyses using large-scale SNP data. *Bioinformatics* 2005; **21**: 3565–3567.

3 Exome Aggregation Consortium, Lek M, Karczewski K, Minikel E, Samocha K, Banks E *et al.* Analysis of protein-coding genetic variation in 60,706 humans. Cold Spring Harbor Labs Journals, 2015 doi:10.1101/030338.

4 Exome Variant Server, NHLBI GO Exome Sequencing Project (ESP), Seattle, WA. 2013; : http://evs.gs.washington.edu/EVS/.

5 Abecasis GR, Altshuler D, Auton A, Brooks LD, Durbin RM, Gibbs RA *et al.* A map of human genome variation from population-scale sequencing. *Nature* 2010; **467**: 1061–73.

6 Consortium TG of the N. Whole-genome sequence variation, population structure and demographic history of the Dutch population. *Nat Genet* 2014; **46**: 818–825.

7 Loohuis LMO, Vorstman JAS, Ori AP, Staats KA, Wang T, Richards AL *et al.* Genome-wide burden of deleterious coding variants increased in schizophrenia. *Nat Commun* 2015; **6**: 7501.

8 Hofman A, Murad SD, Van Duijn CM, Franco OH, Goedegebure A, Arfan Ikram M *et al.* The rotterdam study: 2014 objectives and design update. *Eur J Epidemiol* 2013; **28**: 889–926.

9 Kelley LA, Sternberg MJE. Protein structure prediction on the Web: a case study using the Phyre server. *Nat Protoc* 2009; **4**: 363–71.

10 Zhang Y. I-TASSER server for protein 3D structure prediction. *BMC Bioinformatics* 2008; **9**: 40.

11 Warlich E, Kuehle J, Cantz T, Brugman MH, Maetzig T, Galla M *et al.* Lentiviral Vector Design and Imaging Approaches to Visualize the Early Stages of Cellular Reprogramming. *Mol Ther* 2011; **19**: 782–789.

12 Monaco MCG, Maric D, Bandeian A, Leibovitch E, Yang W, Major EO. Progenitor-derived oligodendrocyte culture system from human fetal brain. *J Vis Exp* 2012. doi:10.3791/4274.

13 Huang GN, Zeng W, Kim JY, Yuan JP, Han L, Muallem S *et al.* STIM1 carboxyl-terminus activates native SOC, I(crac) and TRPC1 channels. *Nat Cell Biol* 2006; **8**: 1003–1010.

14 Stoppini L, Buchs PA, Muller D. A simple method for organotypic cultures of nervous tissue. *J Neurosci Methods* 1991; **37**: 173–82.

15 Najm FJ, Lager AM, Zaremba A, Wyatt K, Caprariello A V, Factor DC *et al.* Transcription factor–mediated reprogramming of fibroblasts to expandable, myelinogenic oligodendrocyte progenitor cells. *Nat Biotechnol* 2013; **31**: 426–433.

16 Koppelmans V, de Groot M, de Ruiter MB, Boogerd W, Seynaeve C, Vernooij MW *et al.* Global and focal white matter integrity in breast cancer survivors 20 years after adjuvant chemotherapy. *Hum Brain Mapp* 2014; **35**: 889–99.

17 Ikram MA, van der Lugt A, Niessen WJ, Krestin GP, Koudstaal PJ, Hofman A *et al.* The Rotterdam Scan Study: design and update up to 2012. *Eur J Epidemiol* 2011; **26**: 811–24.

18 Smith SM, Jenkinson M, Woolrich MW, Beckmann CF, Behrens TEJ, Johansen-Berg H *et al.* Advances in functional and structural MR image analysis and implementation as FSL. In: *NeuroImage*. 2004 doi:10.1016/j.neuroimage.2004.07.051.

19 Smith SM. Fast robust automated brain extraction. *Hum Brain Mapp* 2002; **17**: 143–155.

20 Andersson JLR, Jenkinson M, Smith S. Non-linear registration aka Spatial normalisation FMRIB Technial Report TR07JA2. 2007.

21 Rueckert D, Sonoda LI, Hayes C, Hill DL, Leach MO, Hawkes DJ. Nonrigid registration using free-form deformations: application to breast MR images. *IEEE Trans Med Imaging* 1999; **18**: 712–721.

22 Mori S, Oishi K, Jiang H, Jiang L, Li X, Akhter K *et al.* Stereotaxic white matter atlas based on diffusion tensor imaging in an ICBM template. *Neuroimage* 2008; **40**: 570–582.

23 White T, Schmidt M, Karatekin C. White matter ‘potholes’ in early-onset schizophrenia: a new approach to evaluate white matter microstructure using diffusion tensor imaging. *Psychiatry Res* 2009; **174**: 110–115.

24 Smith SM, Jenkinson M, Johansen-Berg H, Rueckert D, Nichols TE, Mackay CE *et al.* Tract-based spatial statistics: Voxelwise analysis of multi-subject diffusion data. *Neuroimage* 2006; **31**: 1487–1505.

25 White T, Ehrlich S, Ho B, Manoach DS, Caprihan A, Schulz SC *et al.* Spatial characteristics of white matter abnormalities in schizophrenia. *Schizophr Bull* 2013; **39**: 1077–86.

**SUPPLEMENTARY AUTHOR INFORMATION**

**Genetic Risk and Outcome of Psychosis (GROUP) investigators**

R. Bruggeman, MD, PhD, Department of Psychiatry, University Medical Center Groningen, University of Groningen; W. Cahn, MD, PhD, Department of Psychiatry, Rudolf Magnus Institute of Neuroscience, University Medical Center Utrecht; L. de Haan, MD, PhD, Department of Psychiatry, Academic Medical Center, University of Amsterdam; R. S. Kahn, MD, PhD, Department of Psychiatry, Rudolf Magnus Institute of Neuroscience, University Medical Center Utrecht, Utrecht, the Netherlands; C. Meijer, PhD, Department of Psychiatry, Academic Medical Center, University of Amsterdam; I. Myin-Germeys, PhD, South Limburg Mental Health Research and Teaching Network, EURON, Maastricht University Medical Center; J. van Os, MD, PhD, South Limburg Mental Health Research and Teaching Network, EURON, Maastricht University Medical Center, Maastricht, the Netherlands, and King’s College London, King’s Health Partners, Department of Psychosis Studies, Institute of Psychiatry, London, England; and A. A. Bartels-Velthuis, PhD, Department of Psychiatry, University Medical Center Groningen, University of Groningen, Groningen, the Netherlands.

The infrastructure for the GROUP study is funded through the Geestkracht programme of the Dutch Health Research Council (ZON-MW, grant number 10-000-1001), and matching funds from participating pharmaceutical companies (Lundbeck, AstraZeneca, Eli Lilly, Janssen Cilag) and universities and mental health care organizations (Amsterdam: Academic Psychiatric Centre of the Academic Medical Center and the mental health institutions: GGZ Ingeest, Arkin, Dijk en Duin, GGZ Rivierduinen, Erasmus Medical Center, GGZ Noord Holland Noord. Maastricht: Maastricht University Medical Center and the mental health institutions: GGZ Eindhoven en de kempen, GGZ Breburg, GGZ Oost-Brabant, Vincent van Gogh voor Geestelijke Gezondheid, Mondriaan Zorggroep, Prins Clauscentrum Sittard, RIAGG Roermond, Universitair Centrum Sint-Jozef Kortenberg, CAPRI University of Antwerp, PC Ziekeren Sint-Truiden, PZ Sancta Maria Sint-Truiden, GGZ Overpelt, OPZ Rekem. Groningen: University Medical Center Groningen and the mental health institutions: Lentis, GGZ Friesland, GGZ Drenthe, Dimence, Mediant, GGNet Warnsveld, Yulius Dordrecht and Parnassia psycho-medical center (The Hague). Utrecht: University Medical Center Utrecht and the mental health institutions Altrecht, GGZ Centraal, Riagg Amersfoort
