# Supplementary figures and images for: Candidate CSPG4 mutations and induced pluripotent stem cell modeling implicate oligodendrocyte progenitor cell dysfunction in familial schizophrenia
Source: Mol Psychiatry. 2018 Jan 4;24(5):757–71. doi: 10.1038/s41380-017-0004-2 (PMC6755981; doi:10.1038/s41380-017-0004-2)

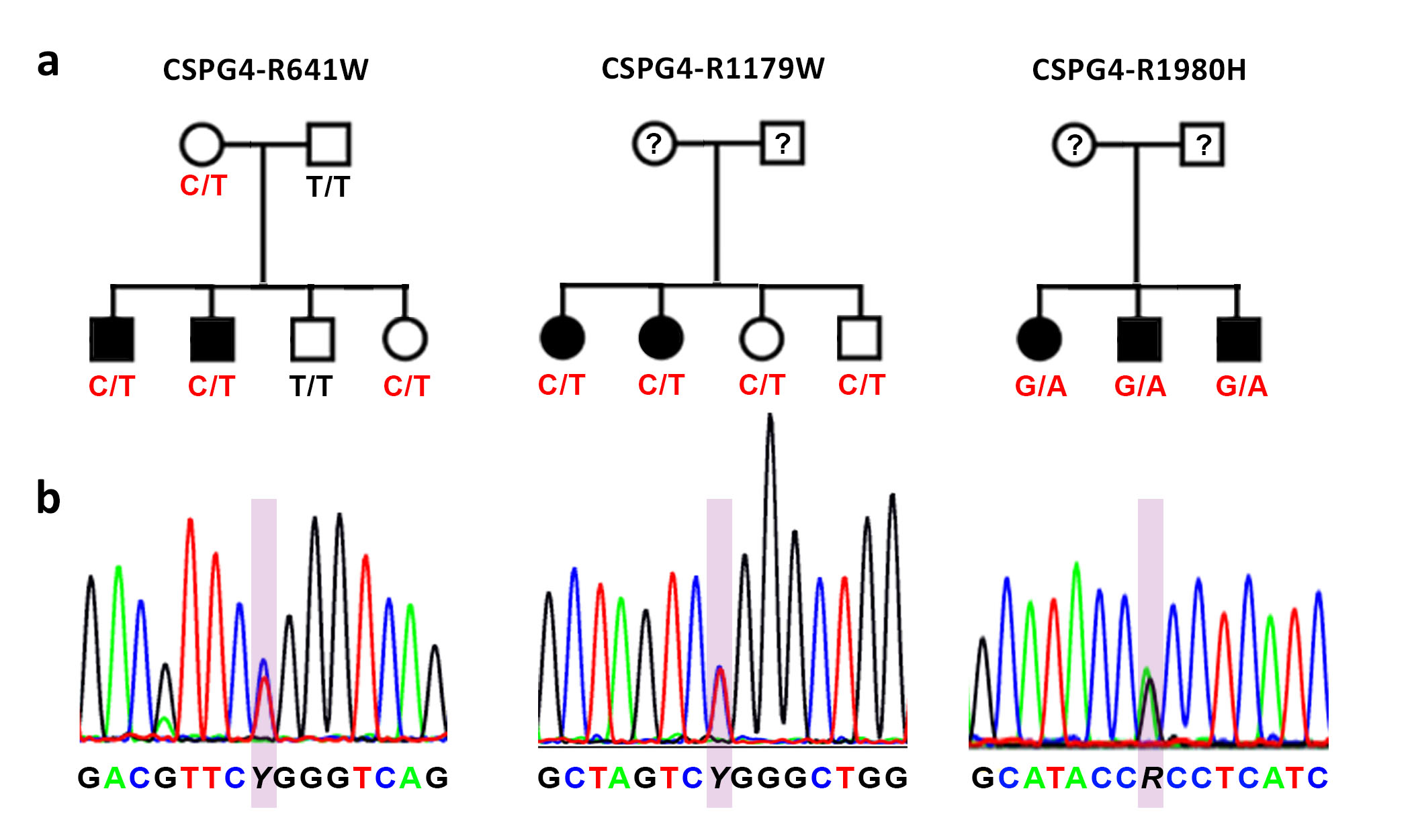

Supplement: Supplementary file 2 — Supplementary Figure 1 [file 41380_2017_4_MOESM2_ESM.jpg]

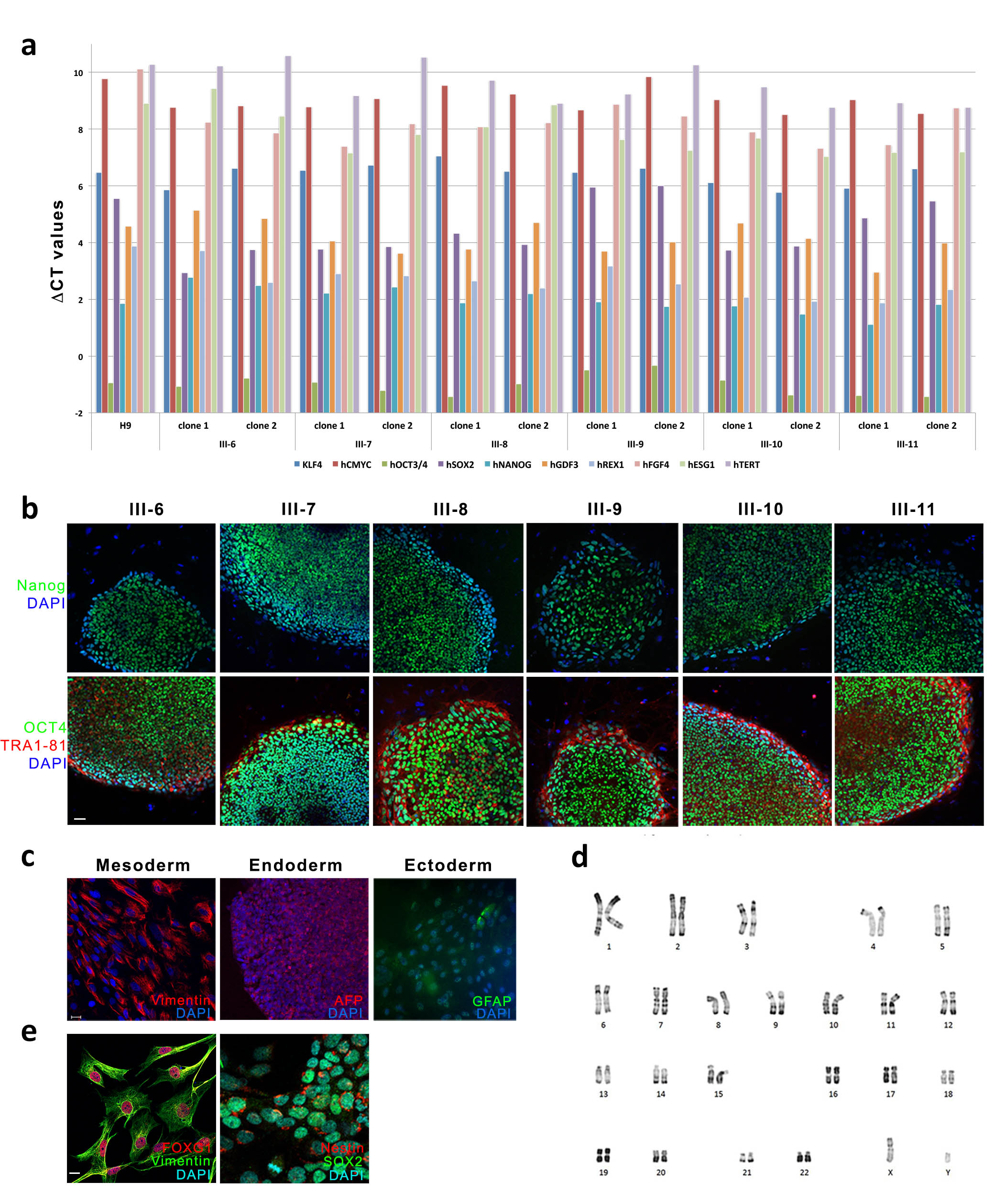

Supplement: Supplementary file 3 — Supplementary Figure 2 [file 41380_2017_4_MOESM3_ESM.jpg]

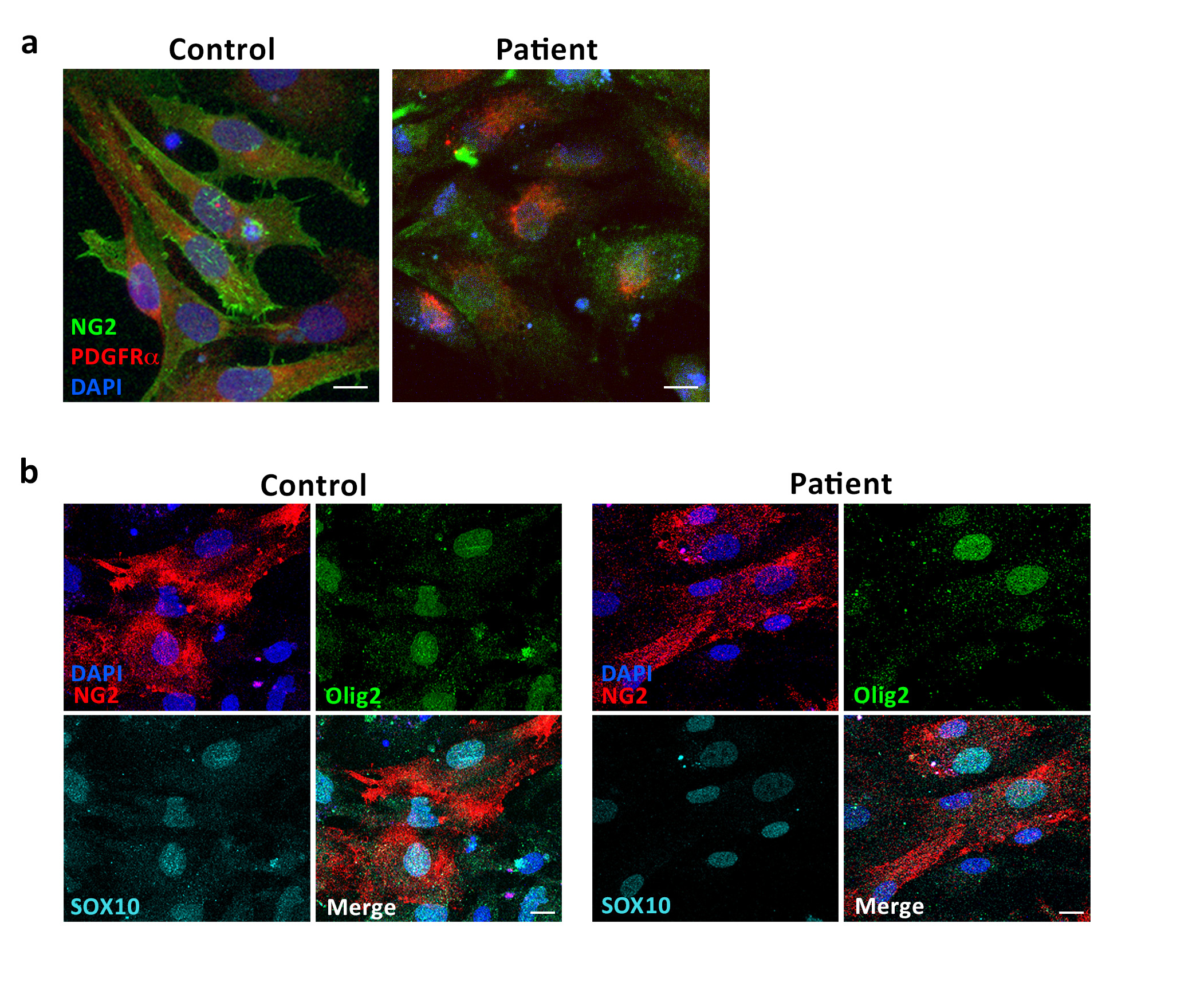

Supplement: Supplementary file 4 — Supplementary Figure 3 [file 41380_2017_4_MOESM4_ESM.jpg]

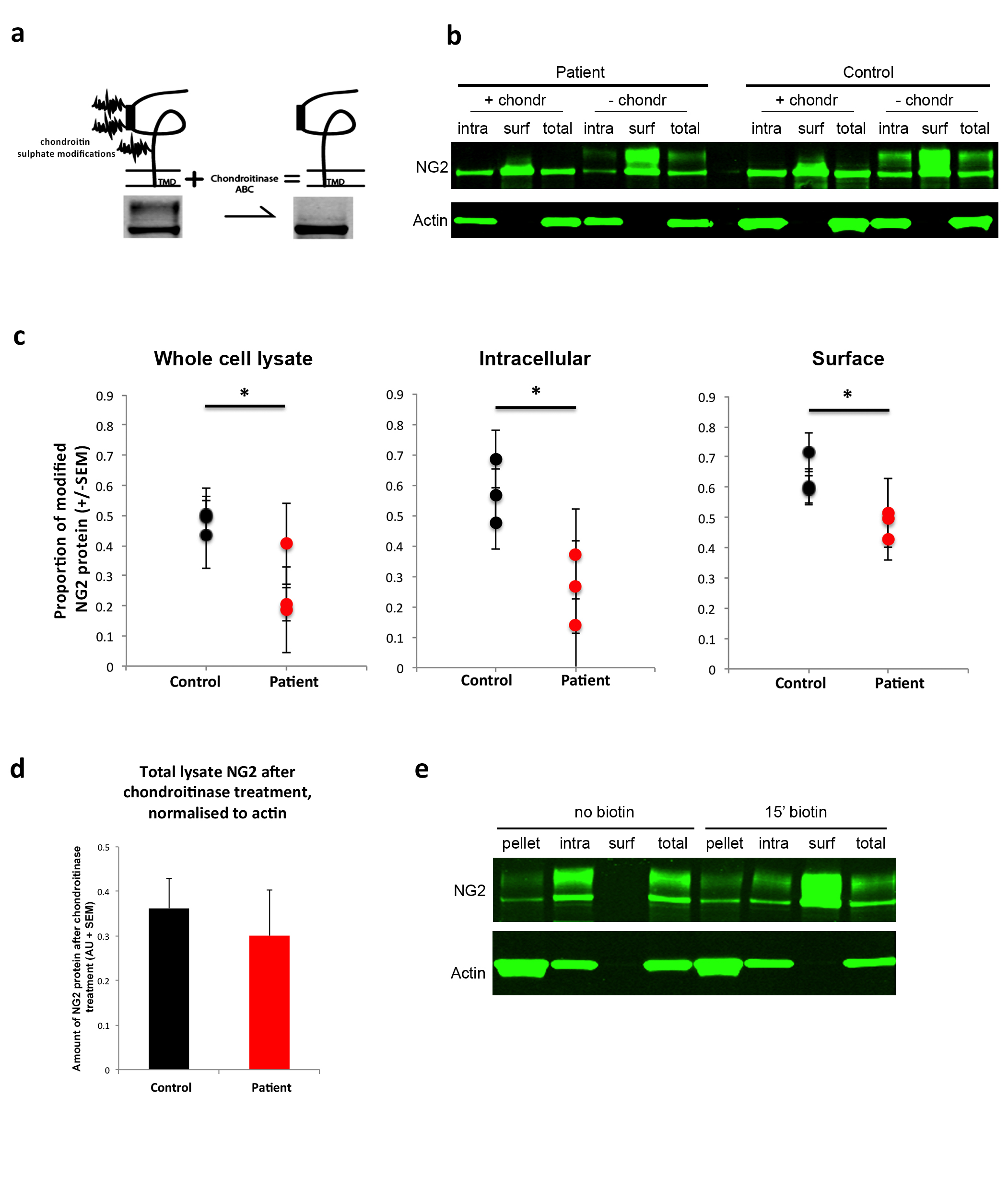

Supplement: Supplementary file 5 — Supplementary Figure 4 [file 41380_2017_4_MOESM5_ESM.jpg]

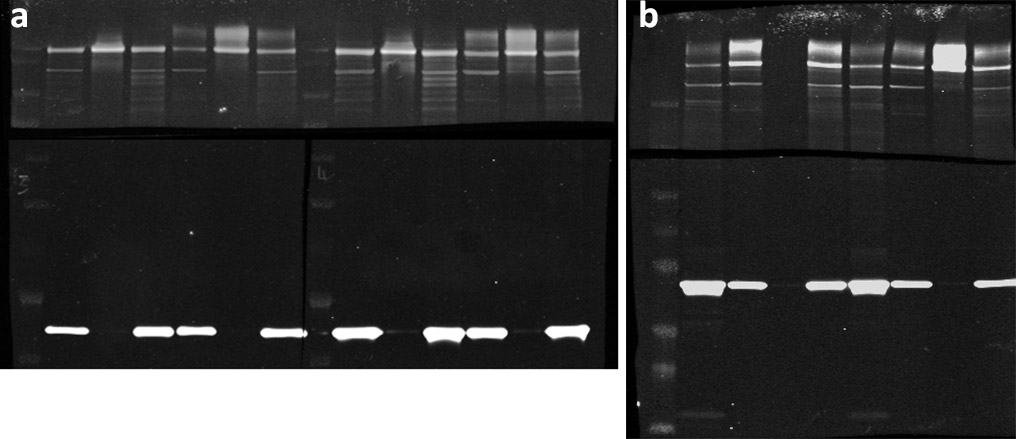

Supplement: Supplementary file 6 — Supplementary Figure 5 [file 41380_2017_4_MOESM6_ESM.jpg]

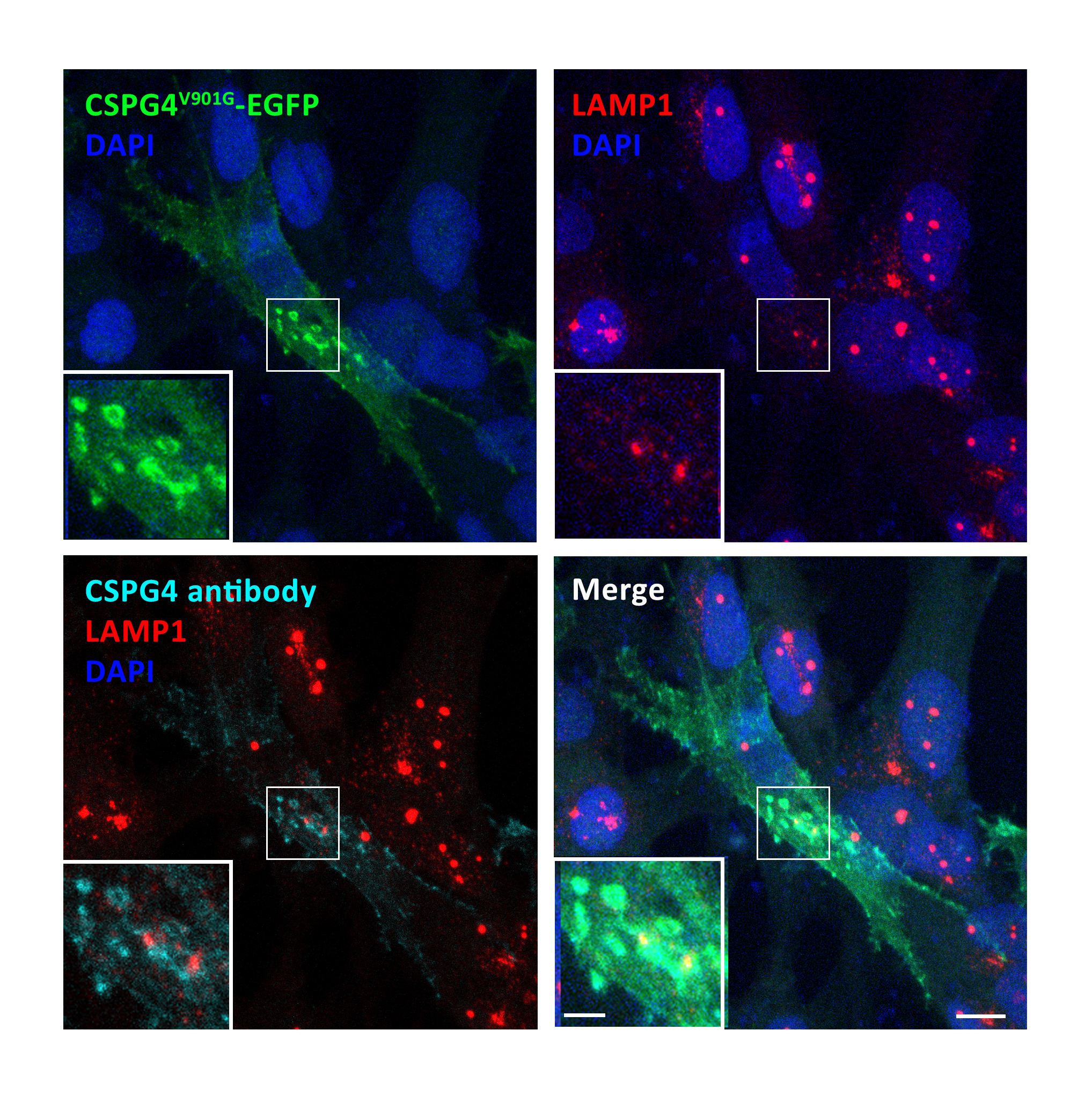

Supplement: Supplementary file 7 — Supplementary Figure 6 [file 41380_2017_4_MOESM7_ESM.jpg]

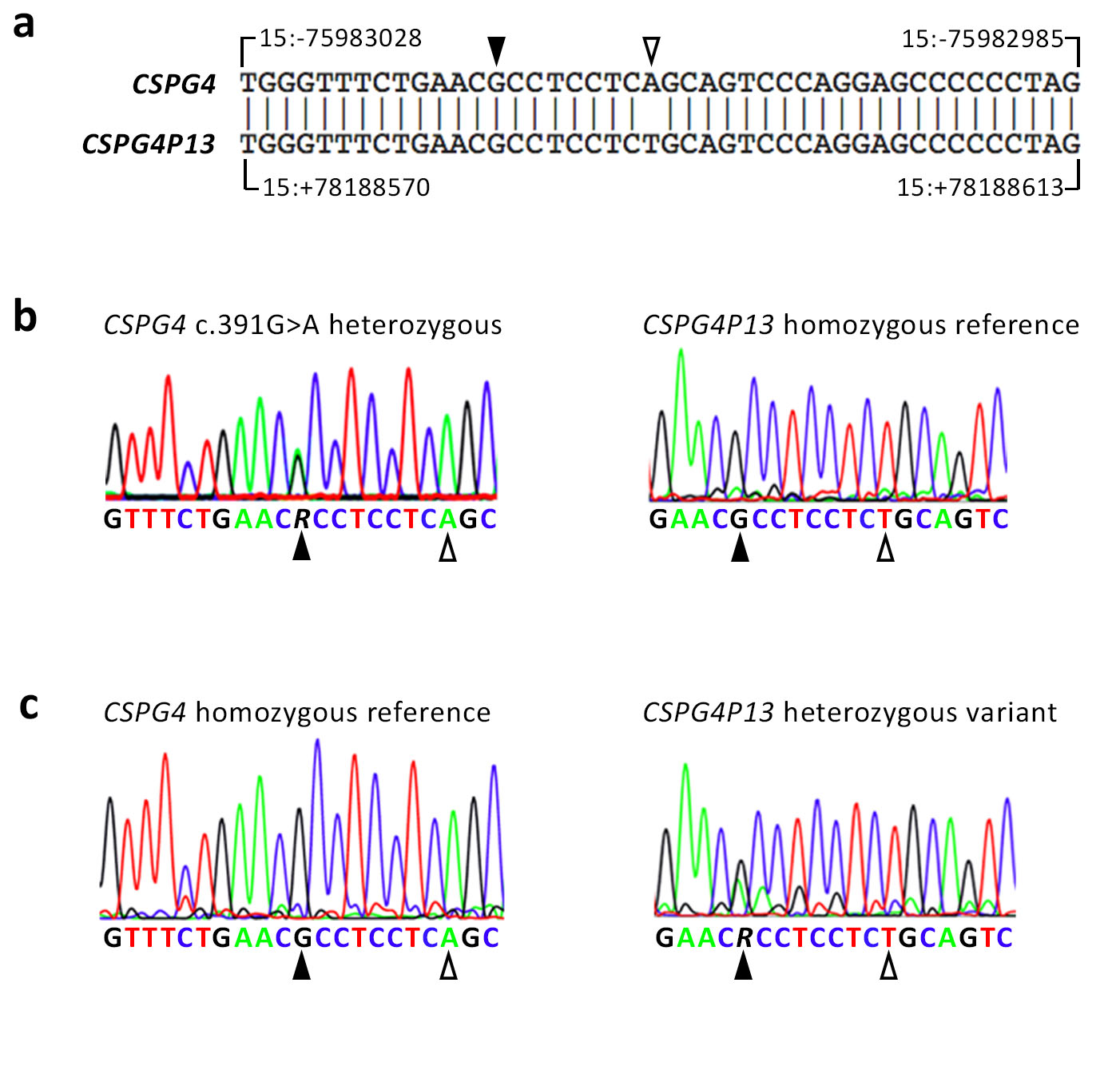

Supplement: Supplementary file 8 — Supplementary Figure 7 [file 41380_2017_4_MOESM8_ESM.jpg]
