# Supplementary material for: Candidate CSPG4 mutations and induced pluripotent stem cell modeling implicate oligodendrocyte progenitor cell dysfunction in familial schizophrenia
Source: Mol Psychiatry. 2018 Jan 4;24(5):757–71. doi: 10.1038/s41380-017-0004-2 (PMC6755981; doi:10.1038/s41380-017-0004-2)
Supplement: Supplementary file 9 — Supplementary Table 1 [file 41380_2017_4_MOESM9_ESM.pdf]

Supplementary Table 1 Discovery Family – Patient characteristics

Patient	II-1	III-5	III-7	III-9	III-11
Diagnosis (DSM)	SZ	SZ	SZ	SZ	SZ
Age of onset first psychosis	46	18	36	18	15
Medication	n/a	Clozapine<br>Biperidene<br>Lithium<br>Oxazepam	Quetiapine<br>Paroxetine<br>Lorazepam	Zuclopenthixol	Zuclopenthixol<br>Trihexyphenidyl<br>Lithium<br>Lorazepam
