# Supplementary material for: Candidate CSPG4 mutations and induced pluripotent stem cell modeling implicate oligodendrocyte progenitor cell dysfunction in familial schizophrenia
Source: Mol Psychiatry. 2018 Jan 4;24(5):757–71. doi: 10.1038/s41380-017-0004-2 (PMC6755981; doi:10.1038/s41380-017-0004-2)
Supplement: Supplementary file 10 — Supplementary Table 2 [file 41380_2017_4_MOESM10_ESM.pdf]

Supplementary Table 2. Linkage analysis – Affected only model

Chromosome	Start position	End position	Start SNP	End SNP	Mb	Max LOD score
2	2686579	17262830	rs11694900	rs17476649	14.576251	0.8809
4	14744996	20524715	rs4464561	rs16869706	5.779719	0.8808
6	164665485	End	rs942731	rs9459964	5.675712	0.8809
7	Start	2739017	rs6583338	rs809547	2.692778	0.8852
7	69935641	97595184	rs4717530	rs9692345	27.659543	0.8852
7	148239179	End	rs6957883	rs1125769	10.809432	0.8852
10	1147045	9247586	rs4880763	rs1469993	8.100541	0.8808
11	Start	4905155	rs1045454	rs11603903	4.700927	0.8852
11	68289796	129160763	rs11228269	rs4644651	60.870967	0.8852
14	89036519	End	rs2116445	rs2583292	18.068524	0.8852
15	39135102	101779863	rs7167406	rs11858464	62.644761	0.8852
16	10480846	76592633	rs7195621	rs12599021	66.111787	0.8826
19	52447068	End	rs8105910	rs7910	6.646396	0.8851
