# Supplementary material for: Candidate CSPG4 mutations and induced pluripotent stem cell modeling implicate oligodendrocyte progenitor cell dysfunction in familial schizophrenia
Source: Mol Psychiatry. 2018 Jan 4;24(5):757–71. doi: 10.1038/s41380-017-0004-2 (PMC6755981; doi:10.1038/s41380-017-0004-2)
Supplement: Supplementary file 11 — Supplementary Table 3 [file 41380_2017_4_MOESM11_ESM.pdf]

Supplementary Table 3. Discovery family candidate variants

Chr	Genomic Position (hg19)	REF	ALT	Gene symbol	Nr Unaffected	MAF ExAC
11	108014748	A	G	ACAT1	1	0.00042
11	113679159	T	C	USP28	1	3.0×10^{-5}
15	75983015	C	T	CSPG4	0	7.8×10^{-5}
16	23080205	C	T	USP31	2	1.6×10^{-5}
16	31371780	T	C	ITGAX	2	4.6×10^{-5}
