# Supplementary material for: Candidate CSPG4 mutations and induced pluripotent stem cell modeling implicate oligodendrocyte progenitor cell dysfunction in familial schizophrenia
Source: Mol Psychiatry. 2018 Jan 4;24(5):757–71. doi: 10.1038/s41380-017-0004-2 (PMC6755981; doi:10.1038/s41380-017-0004-2)
Supplement: Supplementary file 12 — Supplementary Table 4 [file 41380_2017_4_MOESM12_ESM.pdf]

Supplementary Table 4. Sanger sequencing primers for *CSPG4* open reading frame

PCR amplification primers	
**Name**	**Sequence**
CSPG4_ex01_F	ctgccccagagaggaacagc
CSPG4_ex01_R	cccctaactggacagccttgg
CSPG4_ex02_F	gggctggacacaaggtgagc
CSPG4_ex02_R	caagagcctggcagcaagc
CSPG4_ex03a_F	tgccacagcctccaaagtagc
CSPG4_ex03a_R	gcagagtccgggtcataggc
CSPG4_ex03b_F	gctggaggtgtcggtgacg
CSPG4_ex03b_R	ggcacgtgcacacatgtaacc
CSPG4_ex04_F	accagctgcatgtctggctgc
CSPG4_ex04_R	ctggctccgaggagttgtgagg
CSPG4_ex05_F	cagtctgggggttatacacagagagg
CSPG4_ex05_R	gctctgagccgcgaagtagg
CSPG4_ex06-07_F	agctggggccttcctgggta
CSPG4_ex06-07_R	gccaggtccaggcctgtgttt
CSPG4_ex08_F	ggtcacgctgcctctttgc
CSPG4_ex08_R	acgtctgctgccagtgatgc
CSPG4_ex09_F	cccagagtggggcctgag
CSPG4_ex09_R	cccaaccatcaagccaggtc
CSPG4_ex10a_F	gggagggacaatgggagagg
CSPG4_ex10a_R	ccagctcgccagcatctagg
CSPG4_ex10b_F	ctccgggtggtttcagatcg
CSPG4_ex10b_R	tctccaggctcggagtgagc
**Internal Sanger sequencing primers**	
**Name**	**Sequence**
CSPG4_ex3a_int_f1	atgcagccaccctcaatgg
CSPG4_ex3a_int_r1	tcctcctccagcctgcagc
CSPG4_ex3a_int_f2	cgtcacctccaggaacaccg
CSPG4_ex3a_int_r2	ggcagccagagagtgggg
CSPG4_ex3b_int_f1	ctggcccaaggctctgccat
CSPG4_ex3b_int_r1	ggtgccctggcctccttgag
CSPG4_ex3b_int_f2	acaaggctgtcagatggccagg
CSPG4_ex3b_int_f3	ggaggtacggggtgtcttccg
CSPG4_ex3b_int_f4	ccaacctcgacatccgcagtg
CSPG4_ex3b_int_r2	gccggccacgcaacagg
CSPG4_ex3b_int_r3	tgggtgttctgagtgtgcagtgg
CSPG4_ex3b_int_r4	cggcaggagaactcggtcg
CSPG4_ex10a_int_f1	cccagctggctgcagggc
CSPG4_ex10a_int_f2	catcgaggtgcagctgcggg
CSPG4_ex10a_int_r1	ctgccacgctgctcccgttg
CSPG4_ex10a_int_r2	ccggctggggaactgtgtgac
CSPG4_ex10b_int_f1	gaccttgaggacgggaggct
CSPG4_ex10b_int_f2	ctgactgccaagccccgcaa
CSPG4_ex10b_int_r1	gtaaggctcagtggcaaagtcca
CSPG4_ex10b_int_r2	atctaggacggtggggtccagg
