# Supplementary material for: Candidate CSPG4 mutations and induced pluripotent stem cell modeling implicate oligodendrocyte progenitor cell dysfunction in familial schizophrenia
Source: Mol Psychiatry. 2018 Jan 4;24(5):757–71. doi: 10.1038/s41380-017-0004-2 (PMC6755981; doi:10.1038/s41380-017-0004-2)
Supplement: Supplementary file 13 — Supplementary Table 5 [file 41380_2017_4_MOESM13_ESM.pdf]

Supplementary Table 5. qPCR primers for iPSC pluripotency genes

Primer name	Sequence
hOCT3/4-F	GAC AGG GGG AGG GGA GGA GCT AGG
hOCT3/4-R	CTT CCC TCC AAC CAG TTG CCC CAA AC
hSOX2-F	GGG AAA TGG GAG GGG TGC AAA AGA GG
hSOX2-R	TTG CGT GAG TGT GGA TGG GAT TGG TG
hNANOG-F	CAG CCC CGA TTC TTC CAC CAG TCC C
hNANOG-R	CGG AAG ATT CCC AGT CGG GTT CAC C
hGDF3-F	CTT ATG CTA CGT AAA GGA GCT GGG
hGDF3-R	GTG CCA ACC CAG GTC CCG GAA GTT
hREX1-F	CAG ATC CTA AAC AGC TCG CAG AAT
hREX1-R	GCG TAC GCA AAT TAA AGT CCA GA
hFGF4-F	CTA CAA CGC CTA CGA GTC CTA CA
hFGF4-R	GTT GCA CCA GAA AAG TCA GAG TTG
hESG1-F	ATA TCC CGC CGT GGG TGA AAG TTC
hESG1-R	ACT CAG CCA TGG ACT GGA GCA TCC
hTERT-F	CCT GCT CAA GCT GAC TCG ACA CCG TG
hTERT-R	GGA AAA GCT GGC CCT GGG GTG GAG C
hKLF4-F	TGA TTG TAG TGC TTT CTG GCT GGG CTC C
hKLF4-R	ACG ATC GTG GCC CCG GAA AAG GAC C
h-cMYC-F	GCG TCC TGG GAA GGG AGT TCC GGA GC
h-cMYC-R	TTG AGG GGC ATC GTC GCG GGA GGC TG
